# Air Quality Impacts at an E‐Waste Site in Ghana Using Flexible, Moderate‐Cost and Quality‐Assured Measurements

**DOI:** 10.1029/2020GH000247

**Published:** 2020-08-01

**Authors:** Lawrencia Kwarteng, Emmanuel Acquah Baiden, Julius Fobil, John Arko‐Mensah, Thomas Robins, Stuart Batterman

**Affiliations:** ^1^ Department of Biological, Environmental and Occupational Health Sciences University of Ghana Accra Ghana; ^2^ Environmental Health Sciences University of Michigan Ann Arbor Michigan USA

**Keywords:** Air pollution, particulate matter, e‐waste, fires, monitoring

## Abstract

Air quality information is scarce in low‐ and middle‐income countries. This study describes the application of moderate cost approaches that can provide spatial and temporal information on concentrations of particulate matter (PM) needed to assess community and occupational exposures. We evaluated PM levels at the Agbogbloshie e‐waste and scrap yard site in Accra, Ghana, and at upwind and downwind locations, obtaining both optical and gravimetric measurements, local meteorological data and satellite aerosol optical depth. Due to overload issues, the gravimetric 24‐hr samplers were modified for periodic sampling and some optical data were screened for quality assurance. Exceptionally high concentrations (e.g., 1‐hr average PM_10_ exceeding 2000 μg/m^3^) were sometimes encountered near combustion sources, including open fires at the e‐waste site and spoil piles. 24‐hr PM_2.5_ levels averaged 31, 88 and 57 μg/m^3^ at upwind, e‐waste and downwind sites, respectively, and PM_10_ averaged 145, 214 and 190 μg/m^3^, considerably exceeding air quality standards. Upwind levels likely reflected biomass burning that is prevalent in the surrounding informal settlements; levels at the e‐waste and downwind sites also reflected contributions from biomass combustion and traffic. The highest PM levels occurred in evenings, influenced by diurnal changes in emission rates, atmospheric dispersion and wind direction shifts. We demonstrate that moderate cost instrumentation, with some modifications, appropriate data cleaning protocols, and attention to understanding local sources and background levels, can be used to characterize spatial and temporal variation in PM levels in urban and industrial areas.

## Key Points

- Ambient particulate matter was monitored onsite, upwind and downwind of an e‐waste site in Ghana using gravimetric and optical measurements
- E‐waste site emissions increased 24‐hr PM_2.5_ levels by 57 μg/m^3^ over upwind levels of 31 μg/m^3^, and some exceptionally high levels were measured
- Moderate cost methods can measure air quality and source impacts given attention to study design, sampler performance and local influences

## 1 Introduction

Electronic waste (e‐waste) recycling activities include the transport, dismantling, burning, and smelting of electrical and electronic equipment for the purpose of recovering valuable metals, particularly copper and gold. These activities can pose environmental and occupational health and safety concerns, particularly in low and middle income countries (LMICs) and informal settings where controls and inspections are lax or absent (Ackah, 2017; Gangwar et al., 2019; Ohajinwa et al., 2018; Sthiannopkao & Wong, 2013). Recycling activities, particularly burning and smelting, can release significant emissions of airborne pollutants that expose both on‐site workers and the nearby community. Such emissions are highly site‐ and activity‐specific and vary over time, thus, air quality monitoring is needed to assess concentrations and exposures and to determine the specific sources that should be targeted for mitigation. Despite concentrations that can greatly exceed guidelines from WHO and national standards (Djossou et al., 2018; Naidja et al., 2018), air quality monitoring in Africa is scarce, e.g., only 6 of 47 sub‐Saharan countries report PM levels (WHO, 2014). In consequence, the temporal and spatial variation in pollutant concentrations and exposures is poorly characterized. Monitoring plays an essential role in air quality management by documenting exposures and compliance with standards, identifying culpable emission sources, and evaluating the effectiveness of control measures.

Agbogbloshie in central Accra, Ghana has been a hub for large scale e‐waste, automobile and scrap recycling for nearly 15 years. Currently, 4,500–6,000 workers at the site use rudimentary techniques to recover valuable materials (Agyei‐Mensah & Oteng‐Ababio, 2012; Daum et al., 2017). Site activities include delivery and receipt of waste from trucks and carts; sorting and transport of waste to distinct areas for tires, refrigerators, air conditioners, starter motors, televisions, etc.; manual dismantling of some waste types, e.g., hammering‐off aluminum heat exchanger fins, stripping insulation from larger cables using machetes to obtain copper, breaking cathode ray tubes on older televisions to obtain the yoke and its copper windings; open burning of smaller insulated wires, cables and circuit boards to obtain copper; collecting and weighing the recovered metals and other valuables; and transport of products off‐site. Black plumes are frequently seen over the site, primarily originating from two waste burning areas ~350 m apart; fire accelerants used include Styrofoam insulation recovered from refrigerators, tires, and other materials (Amoyaw‐Osei et al., 2011). Workers typically carry out their tasks in groups of two or more without personal protective equipment in small sheds or in the open air (Oteng‐Ababio, 2012). Despite being a signatory to the 1989 Basel Convention that limits transboundary movement of waste and national legislation on e‐waste, Ghana produced an estimated 150,000 tons and imported 215,000 tons of e‐waste in 2009 (Ackah, 2017).

Ambient air quality monitoring at Agbogbloshie and the region has been limited. In addition to monthly and annual PM_10_ concentrations reported by the Ghanaian Environmental Protection Agency (EPA) (Ghana EPA, 2016), we identified seven studies that reported ambient PM concentrations in Ghana over the last 20 years (Aboh et al., 2009; Ahiamadjie, 2017; Arku et al., 2015; Dionisio et al., 2010; Laskaris et al., 2019; Ofosu et al., 2012; Sulemana et al., 2018). While suggesting that PM concentrations can be high, the temporal and spatial coverage of these studies is limited, and diurnal patterns and the impact of e‐waste emissions on community exposure have not been evaluated. The objective of this study is to characterize PM concentrations at the Agbogbloshie e‐waste site and the nearby community, and to present moderate cost sampling methods that enable quality‐assured results. This monitoring forms part of the West Africa‐Michigan Charter II for GEOHealth cohort study, which is analyzing occupational exposures and health risks at this site. Our findings are intended to broaden knowledge on PM levels and exposures at the site, and to provide guidance for air pollution monitoring programs, particularly in LMICs.

## 2 Methods

### 2.1 Site Description

**Figure 1:**
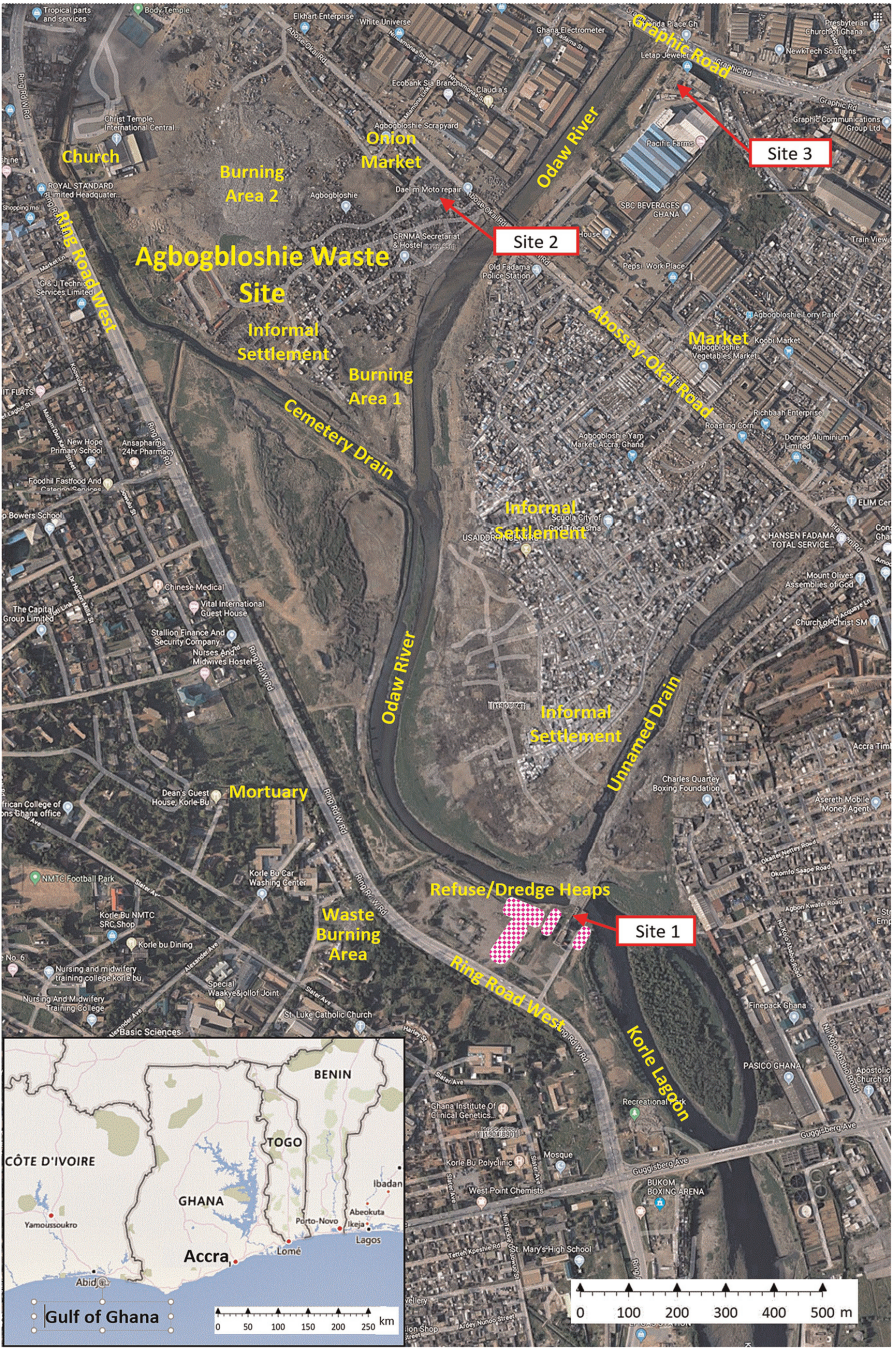
Area map showing e‐waste and air quality monitoring sites.

Agbogbloshie is the informal name for an area about 1 km from central Accra and adjacent to the South Industrial Area that contains the Agbogbloshie e‐waste site and scrap yard. The site is bounded by the Abossey‐Okai Road, the Odaw River, Cemetery Drain, and the Ring Road West. This 0.365 km^2^ area (excluding a large church and recreational fields to the west) is shown in Figure 1. Agbogbloshie is in the Asiedu Keteke sub‐metropolitan area, a commercial hub with ~144,000 residents in a 16 km^2^ area, which lies within Accra, Ghana’s capital, with 2.27 million inhabitants. In addition to the scrap yard and recycling activities, the site contains a health clinic, a technical training center for e‐waste and scrap workers, a football field, mosques, extensive informal housing, workshops for motorcycle, car and electronics repair, commercial cooking, and metal fabrication (e.g., traditional charcoal pots, aluminum cooking pots).

The e‐waste site is adjacent to densely populated residential and commercial areas, including a large market, informal housing to the south, commercial offices and industrial areas to the N and E, vegetable markets to the E, and food markets to the NE. Markets (food, clothing, medicine, furniture, appliances, etc.), commercial cooking, metal working, and banking services are adjacent to the e‐waste site.

The present form of Agbogbloshie is relatively recent. Prior to 2002, an oxbow of the Odaw River cut into the site, the western portion was a waste dump, areas near roads had some informal businesses, and the remainder was largely vacant, based on new reports, historical imagery (Google Earth) and government reports (Awiah, 2017; Ministry of Works and Housing, 2019; Oirere, 2019). For flood control, the river was dredged and channelized and the oxbow drained and filled, and by 2008 the area was established as a hub for large scale e‐waste, automobile and scrap recycling. Informal housing grew throughout the site and gradually reached the southernmost portion where the Odaw River and Cemetery Drain converge. Severe floods in June 2015 led to dredging of the Odaw River and the Korle Lagoon later that year. The river choked again by September 2017 and was subsequently dredged. Another dredging cycle was completed in February 2019, removing over 1 million m^3^ of material; one report suggested that 40,000 m^3^ of silt was deposited annually in the basin (Gambeta, 2019).

### 2.2 Monitoring Approach and Site Description

We utilized “area” or fixed site monitoring with goals of measuring upwind, on‐site and downwind concentrations to understand impacts at the waste site adjusted for upwind or “background” levels, and to assess impacts at downwind locations. Our goal was to collect 24‐hr samples at the three sites simultaneously every sixth day to characterize 1‐hr, 24‐hr and long term PM levels, and to evaluate spatial differences and temporal patterns.

Monitoring site locations were selected by considering e‐waste site activities (especially burning), distance from the e‐waste site, prevailing wind directions, and the ability to obtain electrical power, rain shelter, site security and access, and permissions from property owners and operators. The siting process involved multiple site visits and consultations with leaders of the e‐waste workers, commercial and governmental facilities, and others. We obtained electrical power at two sites and installed a 12 V 80 A‐hr photovoltaic system (LCPC80–12, Jiangsu Oliter Energy Technology Co. Ltd, China) at the upwind site. Site locations are mapped in Figure 1; the supplemental information (SI) provides descriptions, photos and maps of each site (Figures S1‐S3).

At the e‐waste site (site 2), sampling equipment was placed near the ceiling (~2 m height) of a 2‐wall metal shed that served as a meeting area of e‐waste leaders (Figure S1). This shed was ~300 m NNE of burning area 1 and ~300 m E of burning area 2 (Figure 1). This sometimes crowded area had many other sheds and small structures used for weighing, dismantling and storage, and a mosque (within 30 m). We frequently observed individuals cooking, eating, resting, sleeping and selling/buying food, beverages and medicine in this area. The site was 40 m from the busy 2‐lane Abossey‐Okai Road, which fronted considerable commercial activity.

The upwind site (site 1) was 1.35 km SSE of site 2, located at an inoperable pump station/sand filter at the Korle Lagoon (Figure S2). The pump station is a large concrete structure in an open field. Sampling equipment was placed on a concrete shelf on the third level open motor room (~6.5 m above ground level). Nearby land uses include a teaching hospital and mortuary, a dense residential area E of the lagoon, and the 4‐lane Ring Road West, which had intermittent traffic. Fields immediately W of the site had occasional vehicular movement and a few grazing cattle and goats; fields 100–300 m NW were used to transfer rubbish collected by tricycles to trucks for disposal elsewhere, occasional open burning of waste, and football. After establishing the site, we noticed intermittent waste burning at a mortuary 400 m to the W, hidden behind trees lining Ring Road West. In late 2017, the Korle Lagoon and Odaw River adjacent to the site were dredged and spoils (excavated materials) and municipal waste were placed in windrows 3–5 m high in fields immediately SE and W of the pump station. These materials sometimes burned and smoldered; as described later, this resulted in extremely high PM measurements. (After this study, in January 2019, a waste recycling and composting facility for tires, plastic water bags and bottles, metal cans, and organic waste was constructed SW of the pump station.) While site 1 was upwind of the e‐waste site, local activities significantly affected PM levels, and measurements after October 2017 could not be regarded to reflect background levels.

The downwind site (site 3) was 0.48 km NE of the e‐waste site. Monitoring instruments were mounted on a third floor balcony wall (~6 m above the ground) of a 3‐story building (Letap Jewelries Limited Building) used for pharmaceutical production (first two floors) and apartments (third floor; Figures S3–4). This area has considerable commercial activity, a brewery, warehouses, car and truck repair facilities, and it is near a twice‐daily used railway crossing and bridge, the busy 4‐lane Graphic Road, and the normally sluggish and polluted Odaw River channel. In addition to traffic and street merchants on nearby roads, we observed nearby building construction, e‐waste sorting, and occasional open fires on the river’s west bank from the Letap building to the e‐waste site.

### 2.3 PM Instrumentation, Modifications, and Procedures

We monitored PM, temperature and humidity using portable instruments placed in custom fabricated metal “cages” that were open on four sides and locked for safety and security (Figure S4). Integrated 24‐hr PM_2.5_ samples were collected using 47 mm dia pore size 2 μm Teflon filters (SKC, PA, USA), size selective samplers (Impact Sampler, SKC), and personal sampling pumps (Leland Legacy, SKC) operating at 10 L/min. Although the pumps had internal batteries, external power packs were used to allow 24‐h sampling. Near‐continuous measurements (every 1 min) were obtained using 5‐channel optical particle counters (OPCs; Aerocet 831, Met One Instruments, Inc, Oregon, USA) operating at 2.83 L/min. External power also was used with these samplers. Temperature and relative humidity were measured every 1 min using logging instrumentation (UX100–003 data logger; Onset Corporation, Bourne, MA, USA).

Pumps were programmed to sample for a 24‐hr period, and deployment and retrieval of instruments mostly occurred from 10:00 to 16:00. Prior to sampling, new filters were installed, PM_2.5_ pumps were set to 10 L/min using a flowmeter (VFB‐67, Dwyer Instrument Inc, IN, USA) connected to a HEPA capsule filter (Pall Gelman Science, Ann Arbor, MI, USA), and instrument clocks were synchronized. OPC instrument flows were checked and adjusted to 2.83 L/min. After sampling, flow rates were measured again, filters were removed from the sampler cassette using clean forceps, folded in half (exposed side closed), and placed in individual poly bags until weighing. All filters were stored in a clean, sealed container until gravimetric analysis. In the laboratory, filters were weighed before and after sampling using a microbalance (ME‐5, Sartorius, New York, USA) after 48‐hr conditioning at 25 ± 1 C, RH = 33 ± 2%, and deionizing for 30 min. Newly weighed filters were placed in individual labeled poly bags.

Pilot deployments showed that the integrated samplers were significantly overloaded, which was observed visually (Figure S5); overload could result in particle bounce and other issues that would alter sampler performance and the PM size cut. We considered several methods to resolve this problem: a lower flow rate would reduce accumulation, but also change the sampler’s size cut and thus was unacceptable for the selected inlet, and lower flow rates also can increase weighing errors; a shorter sampling period would reduce overload, however, 24‐hr periods were desired to capture both day and night periods; and an elutriator, cyclone or other device upstream of the sampler would exclude coarse PM, but would pose size, pressure drop and cost issues. Instead, we opted to use periodic sampling with a custom‐designed and fabricated system consisting of valves and a programmable cycle timer configured to sample for a 5‐min period and then bypass the sampler for 10 min. This cycle was repeated throughout the 24‐hr sampling period, thus reducing the air volume sampled by two‐thirds. (The system also enabled collection of a second sample on the 10‐min arm, with a volume reduced by one‐third for the cycling described.) Some systems allow automatic cycling of the sampling pump, which also reduces pump wear, but this was not possible with the selected pump. (We will make available the design of the cycle timer upon request to the corresponding author.)

### 2.4 Meteorological and Satellite Data

Local meteorological variables were measured using a weather station (Vantage Pro 2 Precision, Davis Instruments, Hayward, California) placed immediately SSW of site 1 at 4.5 m above ground level (36 m msl), 2.5 m above a ~ 2 m concrete wall that enclosed the sand filter. This site is 1.2 km from the coast (Gulf of Guinea). The surrounding area was largely flat and free of obstructions other than the filter enclosure and dredge spoils heaped in adjacent fields starting late 2017. 1‐min data from the upwind site were collected from 7/30/17 to 12/31/18, although the wind vane sensor failed on 8/28/18. We also obtained 2017–2018 hourly surface observations from Kotoka International Airport (latitude, longitude: 5.605, −0.167; elevation 62.5 m) from NOAA (https://www.ncdc.noaa.gov). The airport site is 12.1 km from the coast and 10.2 km NE of the e‐waste site. Terrain within 900 m of the airport is open; apartments and some midrise commercial buildings are within 1 km.

To help indicate the possible presence of Harmattan dusts, we examined daily maps of aerosol optical depth (AOD) over the region from November 2017 to February 2017 using both the MODIS dark target algorithm and the combined value‐added AOD (CVA‐AOD) (https://earthdata.nasa.gov/earth-observation-data/near-real-time/download-nrt-data/modis-nrt), as described in the SI.

### 2.5 Quality Assurance

Quality assurance (QA) activities included colocation of sampling instruments, use of standard datasheets, and flow checks before and after deployment. For gravimetric samples, we used filter blanks, a minimum of two replicates of gravimetric measurements with an acceptance criterion of 10 μg, confirmation of flow volumes using the pump’s totalizer within 10%, and exclusion of PM masses over 600 mg that indicated an overloaded sampler. Due to power interruptions, sampler problems and field logistics, some sampling periods did not reach 24‐hr; we excluded periods shorter than 75% of the goal (<18 hr). Filters were handled using forceps and powder‐free gloves; different forceps were used for the oiled impactor substrate disc to avoid cross‐contamination. Filter blanks were subjected to same analyses as samples. To assess weighing accuracy, certified 200 mg standards were weighed at beginning and end of each weighing session, and after every 12^th^ filter.

For optical measurements, daily flow and zero checks were performed using a flowmeter connected to a HEPA capsule filter. Colocation tests showed average agreement within 6% for 1‐hr averages of PM_10_ and within 2% for PM_2.5_. While these instruments have a large dynamic range, very high concentrations can produce coincidence error that biases measurements, e.g., multiple small particles appear as a single larger particle that then overestimates mass in larger size channels. There is no specific threshold where coincident error becomes critical (the manufacturer did not provide guidance); our experience with the selected instrument suggests that biases start around 2000 μg/m^3^. Very high humidity also can bias results (as discussed later). Considering the 144,579 1‐min PM_10_ measurements collected, the top 10 values ranged from 4,926 to 11,084 μg/m^3^, 86 measurements (0.06%) exceeded 3,000 μg/m^3^, and 365 (0.25%) exceeded 2000 μg/m^3^. We considered OPC data as potentially biased if PM_10_ exceeded 2000 μg/m^3^. When aggregated to 1‐hr averages, this omitted 7 (0.3%) of the 2,327 hourly averages and dropped the maximum 24‐hr PM_2.5_ average from 865 to 522 μg/m^3^. As discussed later, these exclusions affected only the highest PM measurements. We also checked agreement with the gravimetric measurements, and did not apply correction factors to the optical measurements.

### 2.6 Data Analysis

Hourly concentrations were calculated from OPC data if at least 80% of the 1‐min observations were available and valid, and 24‐hr averages were calculated if at least 80% of hourly averages were available. Analysis focused on PM_2.5_ and PM_10_; the coarse fraction (PM_2.5–10_) was also determined. Optical and gravimetric PM data were compared for the same periods using correlations and scatterplots; again, at least 80% overlap of hourly data were required in these comparisons. We calculated descriptive statistics for the 1‐hr and 24‐hr data and used probability, trend plots with third order polynomial curves to fit the data, and pollution roses to assess distributions, site differences, wind direction, and time‐of‐day patterns. To provide a single “best” estimate of concentration increments over background (site 1) levels, we combined 24‐hr averages from gravimetric and optical instruments, and estimated standard deviations using Gaussian quadrature. Meteorological data was converted to 1‐hr averages, and after ensuring comparability between the sites, the 2‐site average of hourly wind speed and direction was used to obtain a nearly complete meteorological record. Data were converted to the SCRAM format to generate wind roses using WRPLOT (Lakes Environmental, Waterloo, Ontario, Canada) to summarize wind speed and direction statistics.

## 3 Results

### 3.1 Meteorology

Most of the year, this coastal area receives moist maritime air originating over the Atlantic Ocean with little variation in daily temperatures, although cloud cover and rainfall varies. As discussed later, high pressure systems above the Sahara Desert can give rise to dusty Harmattan winds from November to February (Nicholson, 2013). Based on 1‐hr data collected at 2017–8 Kotoka International Airport data, temperatures averaged (± standard deviation) 27.4 ± 2.2 C, relative humidity (RH) averaged 82.2 ± 11.2%, mixing heights averaged 1989 ± 2,701 m (median = 297 m), and precipitation occurred on 166 hours per year (1.9% of hours); precipitation may be underestimated since the record was incomplete (27% of hourly data was missing). Precipitation amounts and trends were similar at the two sites, although hourly and sometimes daily timing of precipitation varied (Figures S6‐S7). The SI discusses daily and monthly trends. Shifts between day (8:00–20:00) and night (20:00–6:00) periods were modest for temperature (28.6–26.2 C), RH (83.8–87.7%), and mixing height (1989–1877 m).

**Figure 2:**
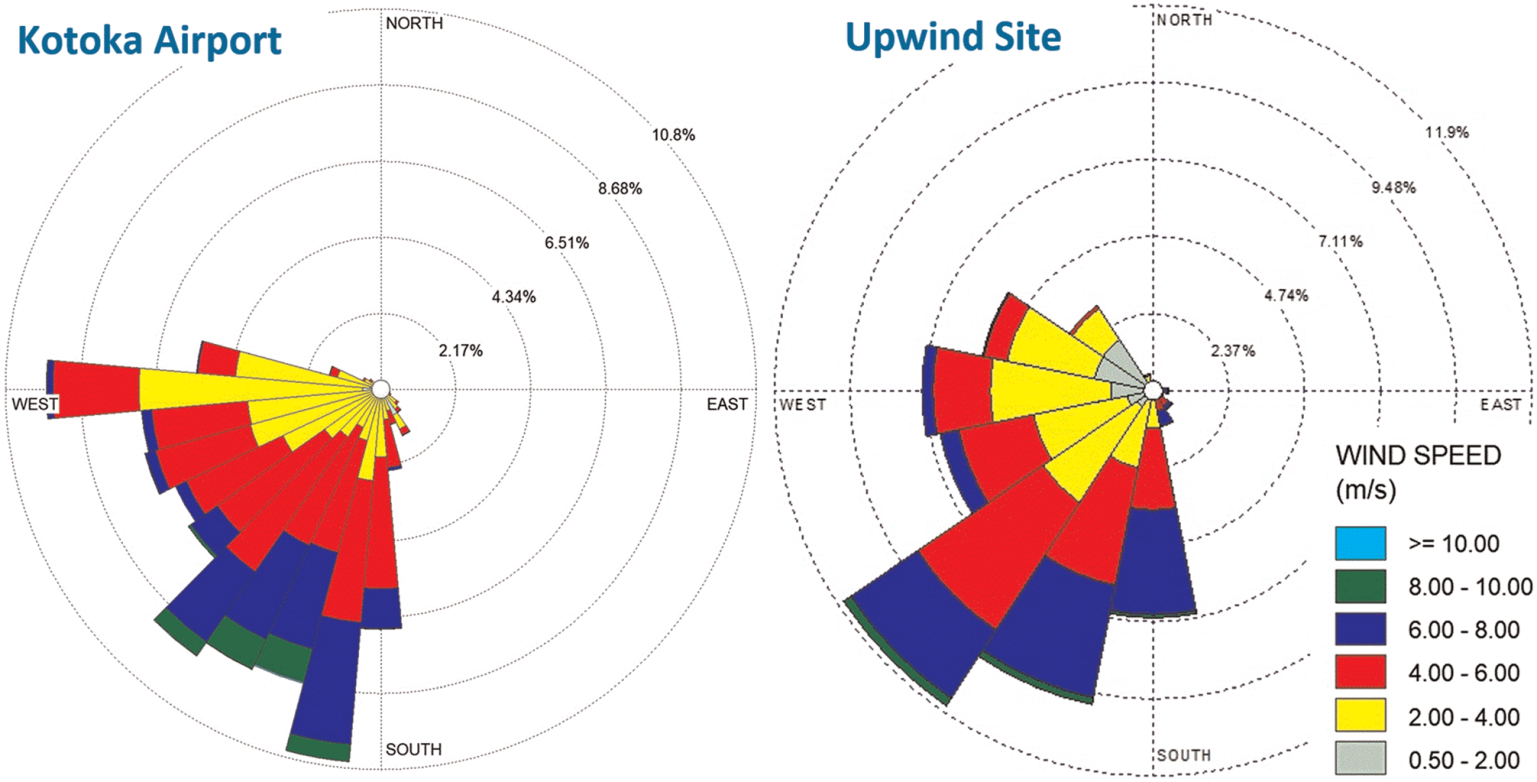
Wind rose at Kotoka airport using hourly data from 2017 and 2018.

Figure 2 shows wind roses for the Kotoka International Airport and the upwind monitoring site; Figures S8‐S9 show diurnal and seasonal roses. Surface winds at the sites are similar, although velocities at the upwind site were slightly lower (3.6 ± 2.3 m/s) and directions more variable, expected given the upwind site’s greater roughness and lower elevation. Winds are predominantly SSW and W; winds from other directions are rare. The airport wind speed (10 m above the ground) averaged 4.4 ± 1.7 m/s and ranged from 1.5–8.2 m/s for the 1^st^ and 99^th^ percentiles. Westerly winds tended to be lighter. Calms (<1 m/s) were rare (<0.6%). The wind field rotates 75‐90^o^ during the day: from midnight to noon, winds are westerly at lower speeds (averaging 3.45 and 4.23 m/s for 0:00–5:00 and 6:00–11:00 periods, respectively); in the afternoon (12:00–17:00), velocity increases (5.62 m/s) and direction transitions to southerly; and in the evening (18:00–23:00), winds return from the SSW and velocity decreases (4.58 m/s). This pattern is highly consistent except from June through August when winds are southwesterly with little diurnal variation and speeds increase (5.04 m/s), due in part to upwelling and cooler water in the Gulf of Ghana that increase the sea‐land temperature differential, and the northernmost movement of the low pressure intertropical convergence zone (ITCZ), which alters the NE trade winds.

In Accra, the consistent wind patterns suggest that plumes from the e‐waste site will disperse N during the afternoon, NE from evening to midnight, and E from midnight to noon. Higher concentrations can occur at night with greater atmospheric stability that decreases dispersion. Later, we show that PM levels at the downwind site (NE of the e‐waste site) increase in the evening, suggesting contributions from the e‐waste site.

### 3.2 Comparison of Gravimetric and Optical Measurements

Of the 99 gravimetric measurements of PM_2.5_ collected, 61 passed QA checks and 43 had corresponding and valid OPC measurements. These 24‐hr average measurements showed reasonable agreement with the optical measurements, e.g., R^2^ = 0.47 (*N* = 43; Figure S10). Previously, the same gravimetric and optical instrumentation (but without periodic sampling) used for 4‐hr shift samples at Agbogbloshie and a local comparison site showed better agreement (R^2^ = 0.75, *N* = 142), probably due to the ability to maintain flows, avoid filter overload with shorter sampling periods, and avoid periods with very high RH (only daytime sampling was used). Calculating the bias between gravimetric and optical measurements as 100% [1 – C_opt_/C_grav_], where C_opt_ and C_grav_ are the average of paired optical and gravimetric measurements, respectively (Williams et al., 2014), optical measurements were 21% low relative to gravimetric measurements, considered as the reference measurement. Importantly, the bias did not change significantly between sites 1–3, which might happen if the optical properties of PM varied at the sites. Because the bias was small and within the expected error range of the gravimetric measurements (due to combined variation in flows, weight determinations, impactor performance, etc.), no correction factor was used for the optical measurements.

High RH may bias optical measurements, particularly for hygroscopic particles, which can demonstrate an exponential increase in hygroscopic growth at high RH, e.g., >85% (Crilley et al., 2018; Di Antonio et al., 2018). OPC responses and light scattering are affected by particle mass, size distribution and composition, including water content (Holstius et al., 2014). Most studies examining RH effects have measured aerosols in industrialized countries where much of the RH artifact is due to rapid growth of the inorganic fraction of the aerosol, e.g., sodium chloride, ammonium nitrate and ammonium sulfate. Among simultaneous 24‐hr gravimetric and optical measurements, we found few divergent cases that could be attributed to high RH; the outstanding case involved concentrations of 120 and 180 μg/m^3^ (gravimetric and optical measurements, respectively) during a 24‐hr period when the RH averaged 92%. Otherwise, screening by humidity did not change agreement between optical and gravimetric measurements, though it is seen in some of the 1‐hr data (discussed below). Potentially a large fraction of PM at Agbogbloshie is organic, suggested by the black color on filters and impaction substrates, the poorly controlled combustion sources, and source apportionments in the literature (described later), and thus is relatively hydrophobic. Our ability to investigate RH biases was constrained since we compared 24‐h PM measurements, and RH stayed in a fairly narrow band.

### 3.3 Highest PM Levels

The highest PM levels in the study occurred at the upwind site for a 12‐hour period starting 23:00 on Jan. 14, 2018 when 1‐hr (optical) PM_2.5_ levels reached 523 μg/m^3^ (average: 369 ± 114 μg/m^3^) and 1‐hr PM_10_ reached 908 μg/m^3^ (665 ± 203 μg/m^3^). These statistics excluded 5 hours with 1‐min PM levels over 2000 μg/m^3^. Including these (potentially biased) data would have increased 1‐hr PM_2.5_ levels to 865 μg/m^3^ (average: 545 ± 223 μg/m^3^), and 1‐hr PM_10_ levels to 2,138 μg/m^3^ (average: 1109 ± 569 μg/m^3^). During this period, QA checks were not met for the corresponding filter‐based measurement (sample U0026). At site 2 during this period, optical measurements were unavailable and the gravimetric measurement (M0043) had flow discrepancies and an overloaded filter. At site 3, levels were fairly typical, e.g., 1‐hr PM_2.5_ levels reached 143 μg/m^3^ (average: 108 ± 15 μg/m^3^) and PM_10_ reached 418 μg/m^3^ (average: 345 ± 34 μg/m^3^), and the gravimetric measurement (L0043) was excluded due to (minor) flow discrepancies; if accepted, the concentration would have been 88 μg/m^3^. The meteorology during this period was not unusual; winds shifted from the SW to the W and wind speeds were low (1.5–3 m/s) during the 5 hours with the highest PM levels, however, RH averaged 89% and reached 94%. Other days with very high PM levels at site 1 showed similar patterns, e.g., on Feb. 8, 2017 from 1:00 to 8:00, 1‐hr PM_2.5_ levels averaged 423 ± 72 μg/m^3^ at site 1; 74 ± 21 at site 2, and 36 ± 11 μg/m^3^ at site 3, and again, winds were light and easterly, and RH was high (average 89% and up to 94%). As discussed later, these high measurements did not occur during Harmattan dust events.

The pattern of nighttime, high and isolated PM events at site 1 with slight E winds is strong evidence of impacts from nearby fires in the spoil piles: most piles were W of the site, fires were sometimes very close (<10 m) to the sampling site, and pile heights approached the sampling height. Time lapse photography at this unoccupied site could have confirmed these emissions, as shown elsewhere (Laskaris et al., 2019). In addition, RH was high during portions of these periods, and site 1 was closest to the coast and possibly experienced higher RH than the airport measurements, suggesting an RH artifact at this site that biased optical measurements upwards.

### 3.4 Hourly and Daily PM Concentrations and Site Differences

**Figure 3:**
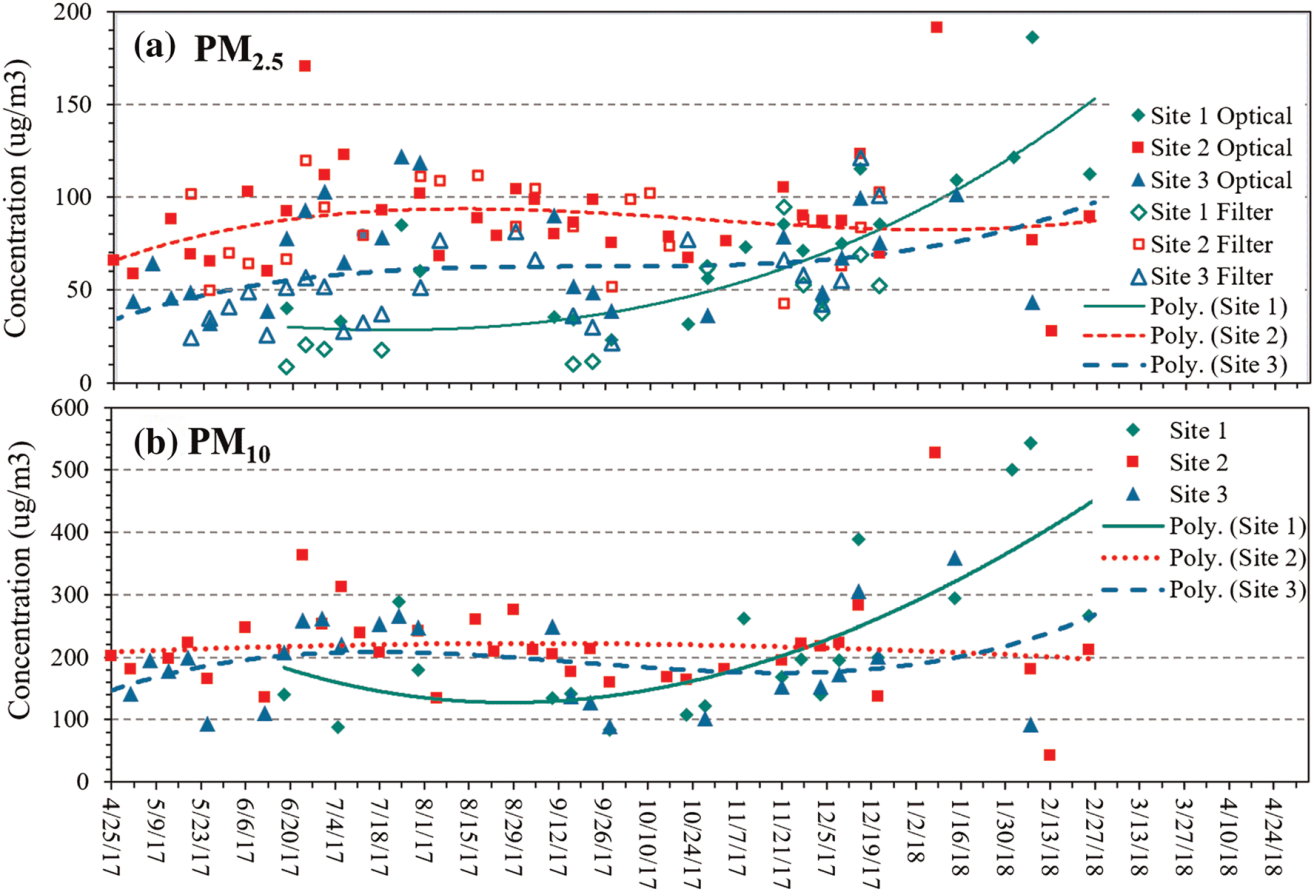
Trends of 24‐hr PM_2.5_ (optical and gravimetric) and PM_10_ (optical) concentrations at the three sites. Curves use 3^rd^ order polynomial. PM_10_ data is based on optical measurements.

**Figure 4:**
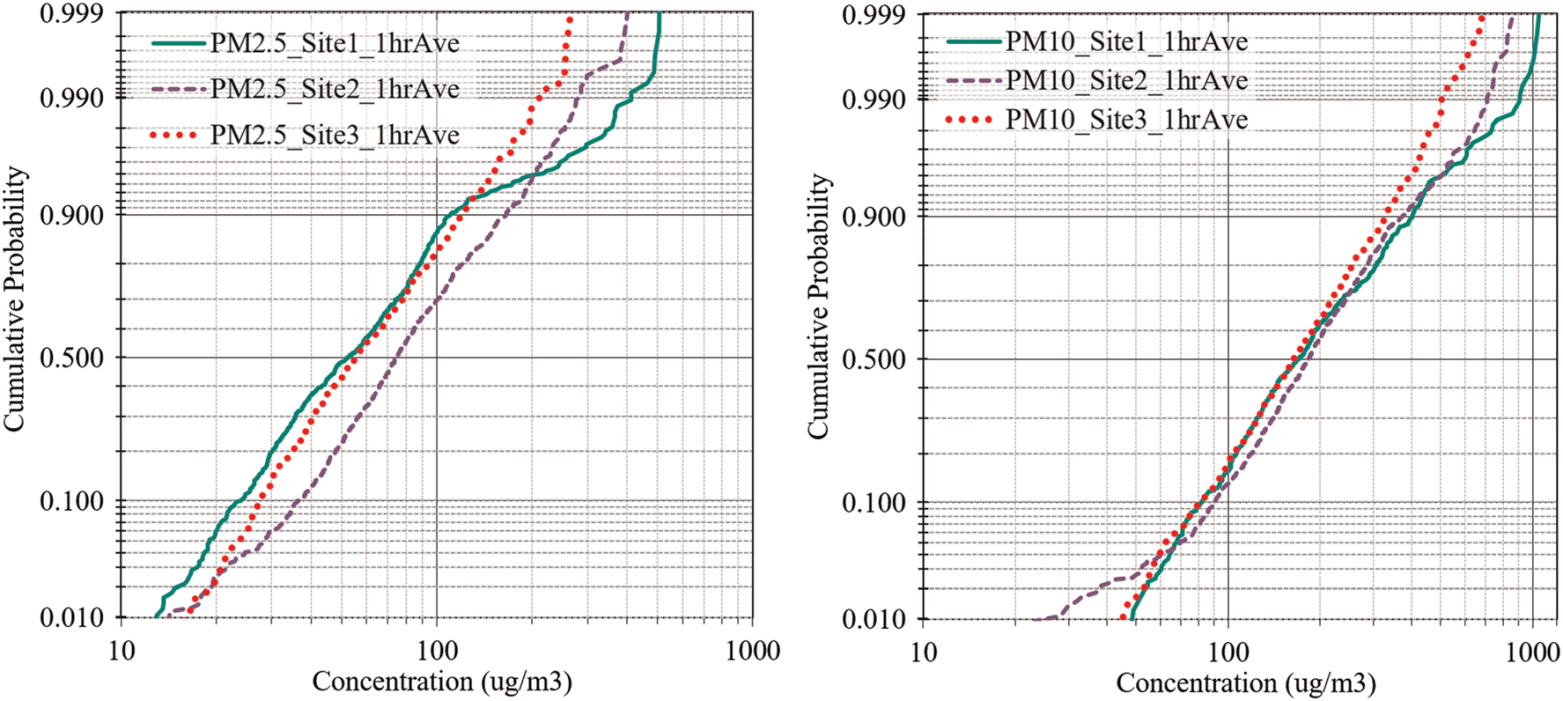
Probability plots of 1‐hr PM_2.5_ and PM_10_ concentrations.

Statistics of the 24‐hr data are presented in Table 1 and trends are plotted in Figure 3. (Table S1 lists statistics of the 1‐hr data.) Across the study, 24‐hr PM_2.5_ levels averaged 63 ± 31 μg/m^3^ (*N* = 61) for the gravimetric data and 79 ± 33 μg/m^3^ (*N* = 81) for the optical data; PM_10_ levels (optical) averaged 210 ± 91 μg/m^3^ (N = 81). Concentrations varied widely, e.g., 1‐hr PM_2.5_ ranged from 7 to 523 μg/m^3^, and 24‐hr levels from 23–192 μg/m^3^ (N = 81). These levels are well over 24‐hr and annual average WHO and Ghanaian standards and guidelines. Probability plots of 1‐hr PM_2.5_ and PM_10_ concentrations suggest lognormal distributions for PM_2.5_ and PM_10_ at sites 2 and 3 (Figure 4). However, at site 1, the distribution’s upper tail is elevated with levels about twice that expected. Up to the 92^nd^ percentile, PM_2.5_ concentrations were lowest at site 1 (upwind) and highest at site 2 (e‐waste); the PM_10_ plot shows that concentrations were similar at the three sites up to the ~90^th^ percentile. While the distributional analysis uses unmatched data and sites have varying number of measurements, it shows the high concentration events that occurred at site 1 after October 2017.

**Table 1:** Summary of 24‐hr average PM_2.5_ and PM_10_ data at the three sites. Matched data shows statistics for days when all three sites have valid data

PM	Sample		2/18/17 to 2/26/18				2/18/17 to 10/27/17				10/28/17–2/26/18			
Size	Type	Statistic	Site1	Site2	Site3	All	Site1	Site2	Site3	All	Site1	Site2	Site3	All
**PM2.5**	Filter‐	Average	38	85	53	63	14	88	46	59	61	78	74	71
	based	St. Dev.	28	21	25	31	5	21	19	33	19	21	30	24
		Min	9	43	22	9	9	50	22	9	38	43	42	38
		Max	95	120	122	122	20	120	81	120	95	103	122	122
		NOBs	12	24	25	61	6	18	19	43	6	6	6	18
	Optical‐	Average	74	90	69	79	43	88	68	74	95	93	69	88
	based	St. Dev.	41	29	27	33	20	24	29	30	38	40	25	36
		Min	23	28	32	23	23	59	32	23	44	28	36	28
		Max	186	191	122	191	85	171	122	171	186	191	102	191
		NOBs	20	36	25	81	8	25	17	50	12	11	8	31
	Combined	Average	60	88	61	72	31	88	57	67	84	88	71	81
	Estimate	St. Dev.	36	26	26	32	14	23	23	31	32	34	27	32
		NOBs	32	60	50	142	14	43	36	93	18	17	14	49
	Concen.	Average	‐	28	0	‐	‐	57	26	‐	‐	4	−12	‐
	Increment	St. Dev.	‐	30	30	‐	‐	21	21	‐	‐	33	30	‐
**PM10**	Optical‐	Average	222	216	191	210	145	214	190	195	273	220	192	233
	based	St. Dev.	129	77	72	91	66	53	63	62	137	118	94	122
		Min	84	43	90	43	84	135	90	84	122	43	92	43
		Max	543	527	359	543	289	364	265	364	543	527	359	543
		NOBs	20	36	25	81	8	25	17	50	12	11	8	31
	Concen.	Average	‐	−6	−31	‐	‐	69	45	‐	‐	−53	−81	‐
	Increment	St. Dev.	‐	99	101	‐	‐	56	64	‐	‐	129	122	‐

The trend plots (Figure 3) show striking and fairly consistent differences in PM_2.5_ levels between the three sites for the period prior to October 26, 2017 when concentrations averaged 31, 88 and 57 μg/m^3^ at sites 1, 2 and 3, respectively (*N* = 14, 43, 36; combined optical/gravimetric estimate; Table 1). Considering upwind site as “background,” the increment over background for PM_2.5_ was 57 ± 21 μg/m^3^ at the e‐waste site (site 2) and 26 ± 21 μg/m^3^ at the downwind site (site 3). PM_2.5–10_ increments were small, 16–18 μg/m^3^, thus differences between sites for PM_10_ were mainly due to PM_2.5_. Increments based on medians or matched data (when all three sites had measurements) were similar. Following October 26, 2017, site 1 levels generally exceeded those at the other two sites, likely reflecting dredging and spoil pile burning discussed earlier. For this later period, modest or negative increments were estimated.

Monitoring height can affect concentration measurements, particularly if monitoring sites are near sources and the atmospheric is stable, which tends to limit vertical mixing. Such effects may be predicted using dispersion models such as AERSCREEN (U.S. EPA, 2016) that represent plume spread which is governed by the vertical dispersion coefficient σ_SC,X_ for stability class SC and downwind distance X. A height change of Δ_H_ (m) changes the concentration by a factor of 1 ‐ exp{− (Δ_H_)^2^/(σ_SC,X_)^2^}. For example, for a distance of 100 m and stable conditions (class F), σ_F,100_ is 2.3 and 7.5 m for rural and urban terrain, respectively; under more common neutral stability (class D), σ_D,100_ is 4.7 and 13.8 m (determined using AERSCREEN). The terrain at Agbogbloshie is urban, thus, the latter dispersion coefficients apply. Monitoring site heights were from 2 to 6.5 m above ground level. A height change of 4 m under stable conditions yields a concentration change of 25% for a source that is 100 m distant (σ_F,100_ = 7.5 m) and 4% for a source that is 300 m distant (σ_F,300_ = 19.9 m). Under neutral stability, changes are 8 and 1%, respectively, for sources 100 and 300 m distant (σ_D,100_ = 13.8 m and σ_D,300_ = 40.2 m). Atmospheric conditions change hourly, and conditions are often unstable and well mixed during the day, which would reduce differences. Site‐specific conditions will determine impacts. At site 1 (upwind), most sources were 100–300 m distant or further, except in late 2017 when smoldering and burning windrows of dredged materials were proximate and nearly at monitoring height (6.5 m) when some of the highest concentrations were observed. At site 2 (e‐waste site), the monitor was 2 m above ground (near breathing height). This site is surrounded by e‐waste activity, e.g., dismantling occurred within 50 m and burning within 300 m; this site would (as was intended) capture high concentrations. Site 3 was ~0.5 km from (most) e‐waste activities, but near a busy road. The monitoring height of 6 m would not significantly decrease concentrations attributable to the e‐waste site, but would likely lower contributions from nearby traffic.

Overall, results indicate that the upwind site functioned as a background site up to October 2017 but not afterwards, that PM_2.5_ at the e‐waste site was highly elevated over background, and that PM_2.5_ at the downwind site was moderately elevated over background. Site differences for PM_10_ were smaller on a relative basis and largely attributable to PM_2.5_.

### 3.5 Diurnal and Wind Sector Variation in PM Levels

**Figure 5:**
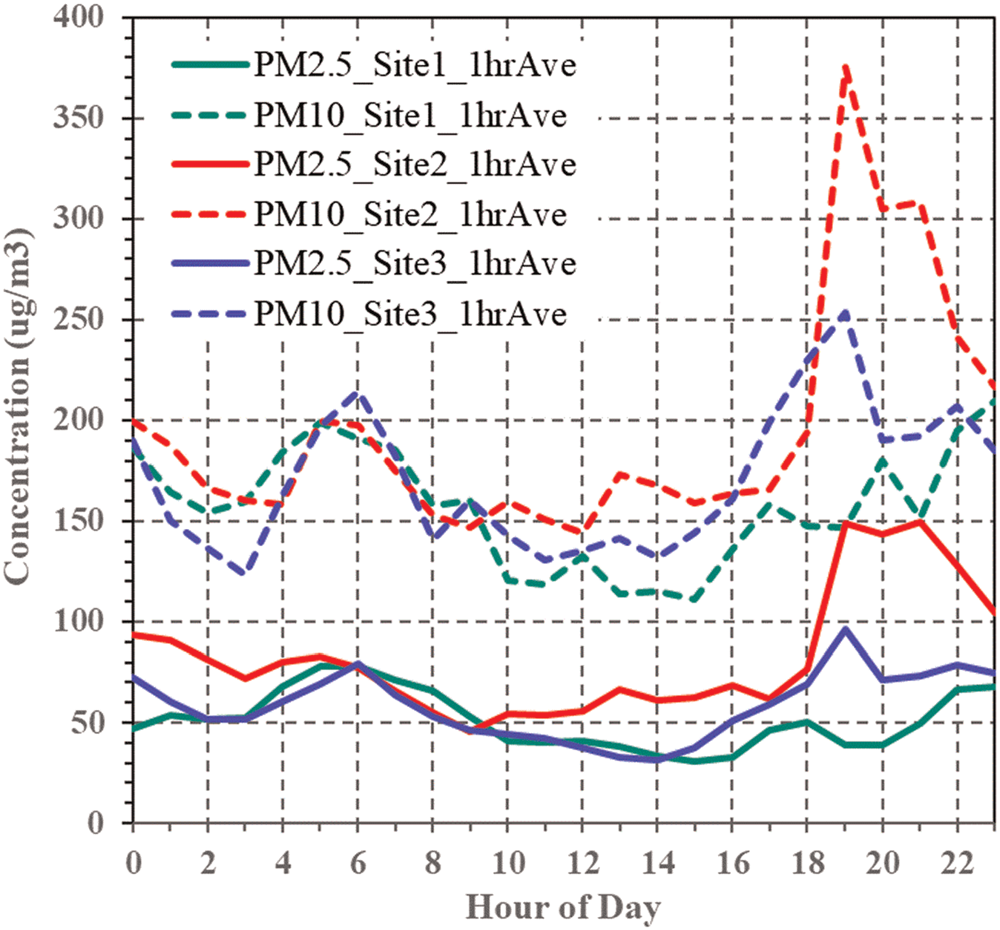
PM_2.5_ and PM_10_ concentrations by time of day. Shows median concentration at each hour. (N = 1,069 at each site.)

Figure 5 displays the diurnal variation of PM levels at each site, using the median hourly concentration to reduce effects of potential outliers. While the sites have some similarities, there are important differences. At site 1, the diurnal pattern was bimodal with a short early morning peak (5:00–7:00) and a prolonged evening peak (17:00–00:00). After October 2017, PM_10_ levels at site 1 were elevated during midday, possibly reflecting dredging activities (Figure S11). At site 2, PM levels peaked in the evening (19:00–22:00) when PM_2.5_ reached nearly 150 μg/m^3^; then gradually declined to 50 μg/m^3^ by mid‐to‐late morning; PM_10_ levels increased somewhat in the early morning (5:00–7:00). As discussed later, prevailing winds shift from the south in the afternoon to the southwest in the evening, which can bring plumes from e‐waste burning in area 1 (which often continues in the evening) and also from cooking using biomass fuels in the extensive informal settlements located south and west of site 2 (Figure S1); in addition, concentrations will increase as the boundary layer height and dispersion are reduced in the evening. At site 3, PM levels rose sharply throughout the evening (17:00–00:00) and a second peak occurred in early morning (5:00–7:00).

**Figure 6:**
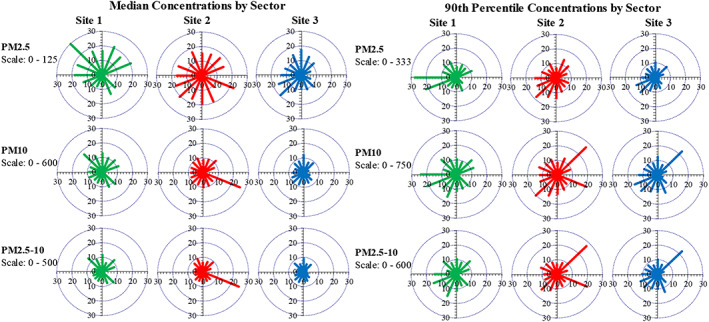
PM_2.5_, PM_10_, and PM_2.5–10_ (coarse fraction) concentrations by wind sector at three sites. Left panel shows median concentration by wind sector. Right panel shows 90^th^ percentile concentrations. Concentration scales differ for each row and set. Scale numbering (0 to 30) is arbitrary.

“Pollutant roses” plotting median and 90^th^ percentile 1‐hr concentrations by wind direction are shown in Figure 6. Because N and E winds were very uncommon, rose “petals” in these directions are unlikely to be representative and should be discounted. At site 1, high concentrations from the NNW suggest e‐waste site emissions, and the high 90^th^ percentile concentrations from the W suggest fires at the adjacent spoil piles. At site 2, median concentrations were approximately uniformly distributed, suggesting emission sources in all directions; 90^th^ percentile levels were highest from the SW (disregarding PM_10_ levels from the NE and E), suggesting burn area 1 or other local activities. At site 3, both median and 90^th^ percentile levels (again neglecting NE and E arms for 90^th^ percentile PM_10_) were highest from the SW, also suggesting e‐waste emissions.

### 3.6 Satellite Data and Harmattan Dusts

The MODIS AOD and CVA‐AOD data are summarized in Table S2, and daily AOD maps are displayed in Figures S12–15. In November and December, 2017, no Harmattan impact is suggested by the satellite or EPA data (Table S2). In 2018, the AOD maps suggests several prolonged and widespread Harmattan dusts episodes over Accra, including Jan. 1–6, possibly on Jan. 8–14, and on Jan. 16–18, Jan. 22–24, Jan. 27–28, Jan. 31‐Feb 2, and Feb. 7–8. We obtained PM measurements on only 3 of these days, and while some levels were high (24‐hr PM_10_ ranged from 92–544 μg/m^3^), Harmattan dusts were not indicated for several reasons: few hours of N to E winds occurred on these days; PM levels or increases across sites were not comparable (Figure 3) as would be expected for regional events; and the highest levels occurred at sites 1 and 2 in short periods that suggest local burning. Possibly, PM levels attributable to Harmattan dusts were too small to discern given the magnitude of local sources near our monitoring sites. As discussed below, earlier studies have shown elevated PM levels during the Harmattan season.

## 4 Discussion

### 4.1 PM Monitoring and Sources

The Ghanaian EPA started monitoring in Accra around 2011 and by 2015, 24‐hr PM_10_ samples were being collected every 6^th^ day at 4–5 “permanent” and 10–11 roadside sites; several sites also measure PM_2.5_. Based on annual reports (Ghana EPA, 2016) and EPA data (E. Apoh, personal communication, Jan. 20, 2020), we list 2015–7 data in Tables S3‐S5. Only monthly summaries were available, and most PM_2.5_ data was for 2015. We first consider the March through November data, i.e., the non‐Harmattan season. In 2015, PM_2.5_ levels at nearby sites averaged 82 μg/m^3^ at the South Industrial Area (SIA) site, located ~1 km N of site 2, and 93 μg/m^3^ at Graphic Road, a roadside site ~460 m ESE of site 3. In 2017, PM_10_ averaged 137 μg/m^3^ at SIA and 237 and 188 μg/m^3^ at Graphic Road for 2015 and 2017, respectively. We obtained comparable levels at sites 2 and 3 (88 and 57 μg/m^3^ for PM_2.5_; 214 and 190 μg/m^3^ for PM_10_), although site 3 was ~62 m upwind (SW) and sheltered from traffic on Graphic Road by a large building, while the EPA site was in the middle of this 4‐lane road (Figure S16). Figure S17 maps 2017 PM_10_ data, and shows some of the highest levels in central Accra and at the e‐waste site. The EPA data also shows higher PM levels during the Harmattan season (December–February) and the highest levels near busy roads, e.g., Weija Junction averaged 155 and 245–295 μg/m^3^ for PM_2.5_ and PM_10_, respectively.

Seven studies were identified that reported PM concentrations in or near Accra; several others examined household pollution from cook stoves (Delapena et al., 2018; Wylie, 2017; Zhou et al., 2011). From April to August 2007, optical PM_2.5_ and PM_10_ measurements collected daily while walking 8–9 km paths over 1 week periods in four neighborhoods had geometric means of 35 and 86 μg/m^3^ in Asylum Down and 21 and 49 μg/m^3^ in Jamestown, the closest neighborhoods to Agbogbloshie; levels were higher along large roads and near roadside biomass fires (trash and stoves) (Dionisio et al., 2010). From January to August 2008, 56 students carrying backpack samplers in the same neighborhoods had 24‐hr geometric mean PM_2.5_ levels from 37–58 μg/m^3^; household fuel use and school location were important determinants (Arku et al., 2015). Ambient levels in these studies may be underestimated due to the lack of evening and nighttime outdoor monitoring. From October to November 2013, 24‐hr PM_10_ (and metal) measurements collected every 6^th^ day at four roadside locations (Weija, Mallam, Kaneshie First Light, Graphic Road, also used by EPA) averaged 167 (Kaneshie) to 228 (Weija) μg/m^3^; the Graphic Road site (along the same road but ~5 km W of our downwind site 3) averaged 182 μg/m^3^ (Sulemana et al., 2018). Lastly, from March 2017 to April 2018, midday 4‐hr shift samples collected from 142 e‐waste workers using backpack monitors and the same optical sensors as in the present study had PM_2.5_ levels averaged 81 μg/m^3^ (Laskaris et al., 2019), similar to our site 2 average of 88 μg/m^3^.

Three studies used receptor modeling to apportion PM sources in Ghana. For a 1‐yr period starting February 2006, gravimetric samples were collected and analyzed for PM, metals and black carbon at Kwabenya, an outlying suburb located ~20 km NE of Accra’s center (Aboh et al., 2009). PM_2.5_ averaged 41 ± 54 μg/m^3^ (median = 26; *N* = 171) and PM_2.5–10_ averaged 138 ± 245 μg/m^3^ (median = 62; N = 171); during Harmattan conditions (defined by Si levels >10 μg/m^3^), PM_2.5_ increased to 97 ± 89 μg/m^3^ (median = 53; *N* = 44) and PM_2.5–10_ to 389 ± 395 μg/m^3^ (median = 182; N = 44); a principal components analysis showed few differences between Harmattan and non‐Harmattan periods, and apportioned PM_2.5_ to crustal (38–39%), vehicles/biomass (24–38%), and industrial (16–33%) sources, while PM_2.5–10_ was apportioned to crustal (41–45%), vehicles/biomass (18–20%), industrial (15–17%), sea spray/other (6–13%), and sand (7%, Harmattan period only) sources. From February 2008 to March 2009, 24‐hr (12‐hr during the Harmattan due to filter overload) samples were collected and analyzed for mass, carbon and metals at Ashaiman, a city (population of 220,000) located ~30 km E of Accra and ~10 km from the industrial port city of Tema; PM_2.5_ averaged 22 μg/m^3^ (range: 6–73; N = 44), and sources identified using positive matrix factorization (PMF) were diesel (18%), soil/dust (18%), gasoline (16%), fresh and aged sea salt (16 and 6%), industry (11%), biomass (9.5%), and 2‐stroke engines (5%) (Ofosu et al., 2012). In dissertation research, PM and metals were measured from May 2010 to April 2011 at a commercial site (trucking depot) ~ 0.5 km NNE of Agbogbloshie (Ahiamadjie, 2017). Median PM_2.5_ and PM_10_ levels were 59 and 107 μg/m^3^ (*N* = 145), respectively; averages were 89 and 138 μg/m^3^; the highest levels occurred during NE Harmattan winds; and sources determined using PMF were Harmattan dusts, e‐waste burning, resuspended soil and dust, industry, vehicles, sea spray, biomass burning and oil burning.

Overall, these studies suggest that average concentrations in urban, industrial or roadside areas in Accra range from 40–106 μg/m^3^ for PM_2.5_ and 91–228 μg/m^3^ for PM_10_ excluding the Harmattan season. These levels are in the range of measurements reported elsewhere in sub‐Saharan Africa, e.g., a 2‐year study at three urban sites in Abidjan, Ivory Coast (423 km W of Accra) and one site in Cotonou, Benin (300 km ENE) using weekly gravimetric sampling at traffic and urban sites reported lower PM_2.5_ levels, averaging 28–32 μg/m^3^, but higher levels, 145 μg/m^3^ in an Abidjan market with ~25 wood‐burning fireplaces that smoked meat and fish or roasted peanuts (Djossou et al., 2018). Such results demonstrate the importance of nearby sources in interpreting monitoring results.

The main PM sources in Accra, as elsewhere in the region, are biomass burning, traffic, industry/energy, and Saharan dust (Aboh et al., 2009; Ahiamadjie, 2017; Naidja et al., 2018; Ofosu et al., 2012; WHO, 2006). Traffic emissions include vehicle exhaust, secondary aerosol formation, and entrained dust from paved and unpaved roads. Additional PM sources include construction activity and sea spray from the Gulf of Guinea. Mechanically‐generated PM, e.g., entrained dust, is predominantly large particles (Pakbin et al., 2010; Rezaei et al., 2018). Importantly, outside of the Harmattan season, combustion sources are responsible for most of the PM_2.5_ (Ahiamadjie, 2017; Ofosu et al., 2012). Biomass burning of waste and wood for cooking is widespread and was observed near each monitoring site, e.g., site 1 had nearby refuse and spoil pile fires (after October 2017); site 2 had nearby and extensive commercial cooking, and biomass fuel use is widespread in the nearby informal housing areas; and site 3 was downwind of occasional fires on the river’s banks. Biomass burning and vehicle exhaust emissions likely form most of the “urban background” found across Accra, including in residential areas. The lowest long‐term PM_2.5_ level in Accra (excluding data from the Harmattan season) was 48 μg/m^3^ measured in a residential area (Danosoman) by EPA in 2015; we measured comparable levels, 44 ± 23 μg/m^3^, at the upwind site. Of note, 24‐hr PM_2.5_ levels at even these sites exceeded the WHO and Ghanaian EPA guidelines (25 and 30 μg/m^3^, respectively). Local impacts due to e‐waste, biomass burning, industry, traffic and Harmattan dusts can be sizeable and add to these levels, further degrading air quality.

### 4.2 PM Attributable to e‐Waste Sites

Several studies have reported PM levels at e‐waste sites. In Moradabad City, India, which has extensive and illegal waste recycling activities, ambient PM_10_ determined gravimetrically during 3 winter months at 3 sites averaged 193–243 μg/m^3^ (Gangwar et al., 2019). In Taizhou, China where a large (1 km^2^) industrial zone recycles ~2 million tons of e‐waste annually, PM_2.5_ measured using high‐volume sampling at two sites 400–500 m distant averaged 38–49 μg/m^3^ (*N* = 7 per site) in summer and 109–154 μg/m^3^ (*N* = 6) in winter (Gu et al., 2010). In Guiyu, China, a community with extensive e‐waste processing, PM_2.5_ samples collected over a 1‐year period averaged 50 μg/m^3^ (*N* = 133), higher than at a reference site (38 μg/m^3^; *N* = 33) (Zheng et al., 2016). Worker exposure estimates based on personal sampling have shown higher concentrations. At Agbogbloshie, the median PM_2.5_ level in shift sampling was 61 μg/m^3^, but tasks like burning resulted in far higher levels (Laskaris et al., 2019). Extremely high levels were reported for workers burning e‐waste in Thailand where PM_2.5_ and PM_10_ levels averaged 2,774 ± 4,173 and 3,215 ± 4,858 μg/m^3^ (N = 33) (Bungadaeng et al., 2019). Indoor PM_10_ levels measured in several e‐waste recycling plants averaged 247–651 μg/m^3^ (Papaoikonomou et al., 2018). These studies note the importance of combustion (especially open burning of waste, circuit boards, wires/cables, Styrofoam, etc.) for fine fraction PM emissions (e.g., PM_2.5_), and the contribution of mechanical processes (dismantling, sorting, shredding, transportation) for coarse PM emissions (e.g., PM_2.5–10_).

The spatial and temporal variability in PM levels is governed by multiple factors. First, some sources have large and regular diurnal changes in emission rates, e.g., traffic associated emissions (vehicle exhaust and road dust) contribute to morning and late afternoon concentration peaks associated with rush‐hour, and cooking using biomass fuels increases in the evening. Other sources have intermittent or irregular emissions, e.g., open burning emissions depend on the fuel, fire stage, wind speed, accelerant, etc. Second, while highly variable, atmospheric dispersion is generally much reduced after sunset as the ground cools (reducing the boundary layer height and mixing), which can greatly elevate concentrations from nearby ground‐level sources (Roy et al., 2012). This was seen at sites 1 and 3, and may have contributed to increases at site 2; however, the diurnal pattern at site 2 (Figure 5) suggest additional factors, e.g., emissions associated with waste handling and possibly cooking. Third, prevailing winds determine impacts downwind of emission sources (Dehghanpour et al., 2014); the regular southerly winds in the afternoon likely decreased PM levels at sites 1 and 3 since few sources were upwind, but increased levels at site 2 due to emissions from burning area 1; evening southwesterly winds brought plumes from burning area 1 to site 3 as seen by a prolonged PM peak; and midnight to noon westerly winds transported emissions from burn area 2 to site 3, and possibly emissions from the mortuary burn area to site 1 (Figure S8). These patterns are consistent and suggest the impact of e‐waste site emissions at site 3. Additional evidence of impacts from Agbogbloshie, up to a distance of up to 2 km (several times longer than to site 3), was suggested by elevated metal concentrations (e.g., Mn, Zn, Cu, Pb) in surface dusts measured along downwind transects (Mudge et al., 2019). We estimate that PM_2.5_ levels at site 3 exceed background levels (at site 1) by 26 μg/m^3^ (Table 1). Much of this increment can be attributed to e‐waste emissions; other sources include traffic on Graphic Road and nearby but infrequent fires. Fourth, background sources, including the regional background and dusty Harmattan winds, discussed next, can affect levels in the region.

### 4.3 PM Attributable to the Harmattan

The Harmattan season is highly variable from year to year and characterized by dry and hazy conditions and little rainfall, which allows widespread entrainment and dispersal of fine and coarse fraction dust from the Sahara Desert across western Africa (Awadzi & Breuning‐Madsen, 2009; Lanzerstorfer, 2017; Toure et al., 2019). In Ghana, these dusts usually originate in intense dust storms (“haboobs”) caused by the Bodélé depression occurring between Tibesti and Lake Chad (K. Sunnu et al., 2018; Naidja et al., 2018). NE Harmattan winds produce white and hazy skies and elevated PM levels in Ghana, typically from December to February. In northern Ghana, the season is dominated by the NE Harmattan winds, and by SW monsoon winds in southern Ghana, although instabilities in the ITCZ can lead to NE Harmattan winds in the south (Awadzi & Breuning‐Madsen, 2009). While only surface PM measurements are directly relevant to community exposures, satellite estimates of AOD and other optical properties, and surface sun photometer estimates of AOD can indicate the potential presence of Harmattan dusts (Toure et al., 2019). These indicators have limitations: the column integrated measures are estimated only during daytime and when clouds do not obscure the sun, MODIS satellite coverage is incomplete at the equator (often the study area is excluded), and most importantly, the relationship between AOD and surface PM concentrations is not direct. While our sampling did not discern impacts of Harmattan events, these events can greatly elevate PM levels (Aboh et al., 2009). For example, 24‐hr PM_2.5_ and PM_10_ levels in Accra reached 350 and 449 μg/m^3^, respectively, in the midst of a severe Harmattan dust event (January 11, 2011) and the PM_2.5_/PM_10_ fraction reached ~80%, compared to 50–60% in non‐Harmattan season (Ahiamadjie, 2017). As noted earlier, EPA data (Tables S3‐S5) also showed higher PM levels during the Harmattan season.

### 4.4 Air Quality Assessment, and Study Strengths and Limitations

This study combined integrated sampling using gravimetric measurements, which are usually required in regulatory and compliance applications, with continuous optical measurements, which are increasingly used in environmental and other applications (Kumar & Gurjar, 2019; Morawska et al., 2018; Mukherjee et al., 2017). The accuracy, precision and reliability of the optical sensors depend on the instrument, PM characteristics (e.g., shape, density and reflectivity) and meteorology (Belosi et al., 2013; Mukherjee et al., 2017; Njalsson & Novosselov, 2018), thus, performance and the need for site‐specific calibrations should be evaluated (Binnig et al., 2007; Walser et al., 2017). As shown by the diurnal and pollution rose plots, the continuous measurements provided by sensors can be highly informative.

Regardless of the instrumentation used, this study shows the importance of an appropriate study design and quality assurance procedures. Our design included: use of both gravimetric and optical sensors to assess the need for correction factors (the gravimetric samples also collected PM for future chemical analyses); use of upwind sites to assess background, e‐waste and downwind impacts; documentation of “microinventories” around monitoring sites that might affect PM levels and interpretations; examination of regional sources and seasonal variation by obtaining sufficient observations and by examining other monitoring and satellite data; collection and analysis of local meteorological information to select monitoring sites and understand collected measurements; and analyses that applied rigorous QA checks, provided appropriate averaging, identified potential outliers, examined temporal, spatial and directional patterns, and used robust statistics.

We recognize limitations of the study. Due to logistical issues, equipment failures, QA checks, and other reasons, our data record had gaps and coverage was incomplete, particularly during the Harmattan season. Upwind, on‐site and downwind levels cannot be fully captured using only three monitoring sites for such a complex environment as Agbogbloshie. The difficulties in establishing appropriate sites should not be underestimated. Continuous monitoring would enable other types of analyses. (The closest known site with continuous hourly or daily PM measurements is in Bamako, Senegal at the US Embassy; the U.S. Embassy in Accra is to start monitoring in 2020). RH measurements at each site might aid detection of RH‐induced artifacts. While perhaps a minor issue in Accra given the very consistent wind patterns, analysis during precipitation events, the Harmattan season, and other periods could be informative. Chemical analysis of the PM, and collection of additional pollutants, would aid source identification.

## 5 Conclusions

We characterized ambient PM levels at the Agbogbloshie e‐waste recycling site and nearby communities using gravimetric and optical instruments and several analysis techniques. Levels at the site were significantly elevated over the background level, which itself was often high, mainly due to biomass burning and vehicle emissions. Very high PM levels, which sometimes overloaded the instruments, typically occurred in the evening due to nearby waste and biomass fires. Overall, we demonstrate that low to moderate cost instrumentation, with some modifications in hardware, appropriate data cleaning, and attention to understanding local sources, meteorology and background levels, can be used to characterize the spatial and temporal variation in PM levels in complex urban and industrial areas. The dearth of air quality information in low‐ and middle‐income countries (LMICs), where pollution levels often far exceed air quality standards, can be addressed using such approaches.

## Conflict of Interest

The authors declare no conflicts of interest relevant to this study.

## Supporting information

Supporting Information S1

## References

[gh2178-bib-0001] Aboh, I. J. K. , Henriksson, D. , Laursen, J. , Lundin, M. , Ofosu, F. G. , Pind, N. , Selin Lindgren, E. , & Wahnström, T. (2009). Identification of aerosol particle sources in semi‐rural area of Kwabenya, near Accra, Ghana, by EDXRF techniques. X‐Ray Spectrometry, 38(4), 348–353. 10.1002/xrs.1172

[gh2178-bib-0002] Ackah, M. (2017). Informal E‐waste recycling in developing countries: review of metal (loid)s pollution, environmental impacts and transport pathways. Environmental Science and Pollution Research, 24(31), 24,092–24,101. 10.1007/s11356-017-0273-y 28944434

[gh2178-bib-0003] Agyei‐Mensah, S. , & Oteng‐Ababio, M. (2012). Perceptions of health and environmental impacts of e‐waste management in Ghana. International Journal of Environmental Health Research, 22(6), 500–517. 10.1080/09603123.2012.667795 22428915

[gh2178-bib-0004] Ahiamadjie, H. (2017). Characterization of Atmospheric Particulate Matter at E‐Waste Landfill Site in Agbogbloshie, Accra. Accra, Ghana: University of Ghana Retrieved from http://ugspace.ug.edu.gh/handle/123456789/26620

[gh2178-bib-0005] Amoyaw‐Osei, Y. , Agyekum, O. O. , Pwamang, J. A. , Mueller, E. , Fasko, R. , & Schluep, M. (2011). Ghana e‐Waste Country Assessment. Retrieved from Accra http://www.basel.int/Portals/4/Basel%20Convention/docs/eWaste/E-wasteAssessmentGhana.pdf

[gh2178-bib-0006] Arku, R. E. , Dionisio, K. L. , Hughes, A. F. , Vallarino, J. , Spengler, J. D. , Castro, M. C. , Agyei‐Mensah, S. , & Ezzati, M. (2015). Personal particulate matter exposures and locations of students in four neighborhoods in Accra, Ghana. Journal of Exposure Science & Environmental Epidemiology, 25(6), 557–566. 10.1038/jes.2014.56 25160763

[gh2178-bib-0007] Awadzi, T. W. , & Breuning‐Madsen, H. (2009). Harmattan dust deposited in Ghana within 2000–2005. West African Journal of Applied Ecology, 11(1). Retrieved from 10.4314/wajae.v11i1.45723

[gh2178-bib-0009] Awiah, D. M. (2017). RCC, AMA , Dredge Masters Initiate Massive de‐Silting of Drains in Accra Ahead of Rains Retrieved from https://www.graphic.com.gh/news/general-news/rcc-ama-dredge-masters-initiate-massive-de-silting-of-drains-in-accra-ahead-of-rains.html

[gh2178-bib-0010] Belosi, F. , Santachiara, G. , & Prodi, F. (2013). Performance evaluation of four commercial optical particle counters. Atmospheric and Climate Sciences, 03(01), 41–46. 10.4236/acs.2013.31006

[gh2178-bib-0011] Binnig, J. , Meyer, J. , & Kasper, G. (2007). Calibration of an optical particle counter to provide mass for well‐defined particle materials. Journal of Aerosol Science, 38(3), 325–332. 10.1016/j.jaerosci.2006.12.001

[gh2178-bib-0012] Bungadaeng, S. , Prueksasit, T. , & Siriwong, W. (2019). Inhalation exposure to respirable particulate matter among workers in relation to their e‐waste open burning activities in Buriram Province, Thailand. Sustainable Environment Research, 29(1), 1–12. 10.1186/s42834-019-0030-7

[gh2178-bib-0013] Crilley, L. R. , Shaw, M. , Pound, R. , Kramer, L. J. , Price, R. , Young, S. , Lewis, A. C. , & Pope, F. D. (2018). Evaluation of a low‐cost optical particle counter (Alphasense OPC‐N2) for ambient air monitoring. Atmospheric Measurement Technology, 11, 709–720. 10.5194/amt-11-709-2018

[gh2178-bib-0014] Daum, K. , Stoler, J. , & Grant, R. J. (2017). Toward a More Sustainable Trajectory for E‐Waste Policy: A Review of a Decade of E‐Waste Research in Accra, Ghana. Internaional Journal of Environmental Research and Public Health, 14(2). 10.3390/ijerph14020135 PMC5334689 28146075

[gh2178-bib-0015] Dehghanpour, A. R. , Halabian, A. H. , & Fallahpour, M. (2014). Impact of wind direction and speed on dusty days. International Journal of Advanced Biological and Biomedical Research, 2(5), 1742–1749. Retrieved from. http://www.ijabbr.com/article_7372_bb5f523872c8d5895902c9a2d74269ea.pdf

[gh2178-bib-0016] Delapena, S. , Piedrahita, R. , Pillarisetti, A. , Garland, C. , Rossanese, M. E. , Johnson, M. , & Pennise, D. (2018). Using personal exposure measurements of particulate matter to estimate health impacts associated with cooking in peri‐urban Accra, Ghana. Energy for Sustainable Development, 45, 190–197. 10.1016/j.esd.2018.05.013

[gh2178-bib-0017] Di Antonio, A. , Popoola, O. A. M. , Ouyang, B. , Saffell, J. , & Jones, R. L. (2018). Developing a relative humidity correction for low‐cost sensors measuring ambient particulate matter. Sensors, 18, 2790 10.3390/s18092790 PMC6164928 30149560

[gh2178-bib-0018] Dionisio, K. L. , Rooney, M. S. , Arku, R. E. , Friedman, A. B. , Hughes, A. F. , Vallarino, J. , Carmichael, H. , Agyei‐Mensah, S. , Spengler, J. D. , & Ezzati, M. (2010). Within‐neighborhood patterns and sources of particle pollution: Mobile monitoring and geographic information system analysis in four communities in Accra, Ghana. Environmental Health Perspectives, 118(5), 607–613. 10.1289/ehp.0901365 20056591 PMC2866674

[gh2178-bib-0019] Djossou, J. , Léon, J. F. , Akpo, A. B. , Liousse, C. , Yoboué, V. , Bedou, M. , Bodjrenou, M. , Chiron, C. , Galy‐Lacaux, C. , Gardrat, E. , Abbey, M. , Keita, S. , Bahino, J. , 'Datchoh, E. , Ossohou, M. , & Awanou, C. N. (2018). Mass concentration, optical depth and carbon composition of particulate matter in the major southern west African cities of Cotonou (Benin) and Abidjan (Côte d'Ivoire). Atmospheric Chemistry and Physics, 18(9), 6275–6291. 10.5194/acp-18-6275-2018

[gh2178-bib-0020] U.S. EPA (2016). AERSCREEN User’s Guide, EPA‐454/B‐16‐004 Office of Air Quality Planning and Standards (pp. 1–115). NC, USA: U.S. Environmental Protection Agency, Research Triangle Park.

[gh2178-bib-0021] Gambeta . (2019). Ghana president Inspects works on Odaw River dredging Retrieved from https://www.gambetanews.com/ghana-president-inspects-works-on-odaw-river-dredging/

[gh2178-bib-0022] Gangwar, C. , Choudhari, R. , Chauhan, A. , Kumar, A. , Singh, A. , & Tripathi, A. (2019). Assessment of air pollution caused by illegal e‐waste burning to evaluate the human health risk. Environment International, 125, 191–199. 10.1016/j.envint.2018.11.051 30721825

[gh2178-bib-0023] Ghana EPA (2016). Annual Report 2015. Retrieved from Accra http://www.epa.gov.gh/epa/sites/default/files/downloads/publications/2015%20Annual%20Report.pdf

[gh2178-bib-0024] Gu, Z. , Feng, J. , Han, W. , Wu, M. , Fu, J. , & Sheng, G. (2010). Characteristics of organic matter in PM2.5 from an e‐waste dismantling area in Taizhou, China. Chemosphere, 80(7), 800–806. 10.1016/j.chemosphere.2010.04.078 20510434

[gh2178-bib-0025] Holstius, D. M. , Pillarisetti, A. , Smith, K. R. , & Seto, E. (2014). Field calibrations of a low‐cost aerosol sensor at a regulatory monitoring site in California. Atmospheric Measurements Technology, 7, 1121–1131. 10.5194/amt-7-1121-2014

[gh2178-bib-0026] Kumar, A. , & Gurjar, B. R. (2019). Low‐cost sensors for air quality monitoring in developing countries – A critical view. Asian Journal of Water, Environment and Pollution, 16(2), 65–70. 10.3233/ajw190021

[gh2178-bib-0027] Lanzerstorfer, C. (2017). Variations in the composition of house dust by particle size. Journal of Environmental Science and Health. Part A, Toxic/Hazardous Substances & Environmental Engineering, 52(8), 770–777. 10.1080/10934529.2017.1303316 28394695

[gh2178-bib-0028] Laskaris, Z. , Milando, C. , Batterman, S. , Mukherjee, B. , Basu, N. , O'Neill, M. , Robins, T. G. , & Fobil, J. N. (2019). Derivation of time‐activity data using wearable cameras and measures of personal inhalation exposure among Workers at an Informal Electronic‐Waste Recovery Site in Ghana. Ann Work Expo Health, 63(8), 829–841. 10.1093/annweh/wxz056 31334545 PMC6788341

[gh2178-bib-0029] Ministry of Works and Housing . (2019). Greater Accra Resilent and Integrated development project (GARID): The environmental Impact Assessment [EIA] study for dredging inn the Odaw Basin Retrieved from Accra http://documents.worldbank.org/curated/en/475521551417349814/pdf/SFG4914-V3-EA-REVISED-P164330-PUBLIC-Disclosed-2-28-2019.pdf

[gh2178-bib-0030] Morawska, L. , Thai, P. K. , Liu, X. , Asumadu‐Sakyi, A. , Ayoko, G. , Bartonova, A. , Bedini, A. , Chai, F. , Christensen, B. , Dunbabin, M. , Gao, J. , Hagler, G. S. W. , Jayaratne, R. , Kumar, P. , Lau, A. K. H. , Louie, P. K. K. , Mazaheri, M. , Ning, Z. , Motta, N. , Mullins, B. , Rahman, M. M. , Ristovski, Z. , Shafiei, M. , Tjondronegoro, D. , Westerdahl, D. , & Williams, R. (2018). Applications of low‐cost sensing technologies for air quality monitoring and exposure assessment: How far have they gone? Environment International, 116, 286–299. 10.1016/j.envint.2018.04.018 29704807 PMC6145068

[gh2178-bib-0031] Mudge, S. M. , Pfaffhuber, K. A. , Fobil, J. N. , Bouman, E. A. , Uggerud, H. T. , & Thorne, R. J. (2019). Using elemental analyses and multivariate statistics to identify the off‐site dispersion from informal e‐waste processing. Environmental Science: Processes & Impacts, 21(12), 2042–2057. 10.1039/C9EM00444K 31693034

[gh2178-bib-0032] Mukherjee, A. , Stanton, L. G. , Graham, A. R. , & Roberts, P. T. (2017). Assessing the utility of low‐cost particulate matter sensors over a 12‐week period in the Cuyama Valley of California. Sensors (Basel), 17(8). 10.3390/s17081805 PMC5579502 28783065

[gh2178-bib-0033] Naidja, L. , Ali‐Khodja, H. , & Khardi, S. (2018). Sources and levels of particulate matter in north African and sub‐Saharan cities: A literature review. Environmental Science and Pollution Research International, 25(13), 12,303–12,328. 10.1007/s11356-018-1715-x 29557037

[gh2178-bib-0034] Nicholson, S. E. (2013). The west African Sahel: A review of recent studies on the rainfall regime and its interannual variability. ISRN Meteorology, 2013, 1–32. 10.1155/2013/453521

[gh2178-bib-0035] Njalsson, T. , & Novosselov, I. (2018). Design and optimization of a compact low‐cost optical particle Sizer. Journal of Aerosol Science, 119, 1–12. 10.1016/j.jaerosci.2018.01.003 30270936 PMC6159267

[gh2178-bib-0036] Ofosu, F. G. , Hopke, P. K. , Aboh, I. J. K. , & Bamford, S. A. (2012). Characterization of fine particulate sources at Ashaiman in Greater Accra, Ghana. Atmospheric Pollution Research, 3(3), 301–310. 10.5094/apr.2012.033

[gh2178-bib-0037] Ohajinwa, C. M. , van Bodegom, P. M. , Vijver, M. G. , Olumide, A. O. , Osibanjo, O. , & Peijnenburg, W. (2018). Prevalence and injury patterns among electronic waste workers in the informal sector in Nigeria. Injury Prevention, 24(3), 185–192. 10.1136/injuryprev-2016-042265 28679520

[gh2178-bib-0038] Oirere, S. (2019). Dredging to control flooding in Ghana’s Accra region. DPC October Edition. Retrieved from https://dredgingandports.com/news/news-dredging/2019/dredging-to-control-flooding-in-ghana/

[gh2178-bib-0039] Oteng‐Ababio, M. (2012). Electronic Waste Management in Ghana ‐ Issues and Practices In Sustainable Development ‐ Authoritative and Leading Edge Content for Environmental Management (Chapter 7). Rijeka, Croatia: InTech.

[gh2178-bib-0040] Pakbin, P. , Hudda, N. , Cheung, K. L. , Moore, K. F. , & Sioutas, C. (2010). Spatial and temporal variability of coarse (PM10−2.5) particulate matter concentrations in the Los Angeles area. Aerosol Science and Technology, 44(7), 514–525. 10.1080/02786821003749509

[gh2178-bib-0041] Papaoikonomou, K. , Emmanouil, C. , Vasilato, V. , Diapouli, E. , Grigoratos, T. , Zafirakou, A. , & Kungolos, A. (2018). PM10 and elemental concentrations in a dismantling Plant for Waste of electrical and electronic equipment in Greece. Aerosol and Air Quality Research, 18(6), 1457–1469. 10.4209/aaqr.2017.12.0557

[gh2178-bib-0042] Rezaei, S. , Naddafi, K. , Hassanvand, M. S. , Nabizadeh, R. , Yunesian, M. , Ghanbarian, M. , Atafar, Z. , Faraji, M. , Nazmara, S. , Mahmoudi, B. , Ghozikali, M. G. , Ghanbarian, M. , & Gholampour, A. (2018). Physiochemical characteristics and oxidative potential of ambient air particulate matter (PM10) during dust and non‐dust storm events: A case study in Tehran, Iran. Journal of Environmental Health Science and Engineering, 16(2), 147–158. 10.1007/s40201-018-0303-9 30728987 PMC6277329

[gh2178-bib-0043] Roy, S. , Gupta, P. , & Nath Singh, T. (2012). Studies on meteorological parameters and mixing height in gold mining area. Resources and Environment, 2(5), 228–239. 10.5923/j.re.20120205.06

[gh2178-bib-0044] Sthiannopkao, S. , & Wong, M. H. (2013). Handling e‐waste in developed and developing countries: Initiatives, practices, and consequences. Science of the Total Environment, 463‐464, 1147–1153. 10.1016/j.scitotenv.2012.06.088 22858354

[gh2178-bib-0045] Sulemana, R. , Boohene, M. , & Sossou, K. B. (2018). Assessment of heavy metal concentrations in particulate matter (PM10) in the ambient air of selected roadsides in the Accra Metropolis of Ghana, West Africa. Journal of Applied Thought, 6(1), 35–54. Retrieved from. https://www.researchgate.net/publication/330840478_Assessment_of_Heavy_Metal_Concentrations_in_Particulate_Matter_PM10_in_the_Ambient_Air_of_Selected_Roadsides_in_the_Accra_Metropolis_of_Ghana_West_Africa

[gh2178-bib-0046] Sunnu, K. , Afeti, G. , & Resch, F. (2018). Daily Levels of the Harmattan Dust near the Gulf of Guinea over 15 Years: 1996–2011. Environment and Ecology Research, 6(6), 593–604. 10.13189/eer.2018.060609

[gh2178-bib-0047] Toure, N. O. , Gueye, N. R. D. , Mbow‐Diokhane, A. , Jenkins, G. S. , Li, M. , Drame, M. S. , Coker, K. A. R. , & Thiam, K. (2019). Observed and Modeled seasonal air quality and respiratory health in Senegal during 2015 and 2016. GeoHealth, 3, 423–442. 10.1029/2019GH000214 32159028 PMC7038905

[gh2178-bib-0048] Walser, A. , Sauer, D. , Spanu, A. , Gasteiger, J. , & Weinzierl, B. (2017). On the parametrization of optical particle counter response including instrument‐induced broadening of size spectra and a self‐consistent evaluation of calibration measurements. Atmospheric Measurement Techniques, 10(11), 4341–4361. 10.5194/amt-10-4341-2017

[gh2178-bib-0049] WHO . (2006). Air Quality Guidelines: Global Update 2005. Retrieved from Copenhagen: https://apps.who.int/iris/bitstream/handle/10665/107823/E90038.pdf?sequence=1&isAllowed=y

[gh2178-bib-0050] WHO . (2014). WHO’s Ambient Air Pollution Database‐Update 2014. Retrieved from Geneva: https://www.who.int/phe/health_topics/outdoorair/databases/AAP_database_results_2014.pdf

[gh2178-bib-0051] Williams, R. , Kilaru, V. , Snyder, E. , Kaufman, A. , Dye, T. , Rutter, A. , Russell, A. , & Hafner, H. (2014). Air Sensor Guidebook. NC, USA: National Exposure Research Laboratory, U.S. Environmental Protection Agency Retrieved from https://cfpub.epa.gov/si/si_public_record_Report.cfm? Lab=NERL&dirEntryId=277996#:~:text=Description%3A,technologies%20for%20air%20quality%20measurements.

[gh2178-bib-0052] Wylie, B. J. (2017). 31: The Ghana randomized air pollution and health study (GRAPHS): A cluster‐randomized trial of clean cookstoves to improve obstetric outcomes. American Journal of Obstetrics and Gynecology, 216(1). 10.1016/j.ajog.2016.11.923

[gh2178-bib-0053] Zheng, X. , Xu, X. , Yekeen, T. A. , Zhang, Y. , Chen, A. , Kim, S. S. , Dietrich, K. N. , Ho, S.‐M. , Lee, S.‐A. , Reponen, T. , & Huo, X. (2016). Ambient air heavy metals in PM_2.5_ and potential human health risk assessment in an informal electronic‐waste recycling site of China. Aerosol and Air Quality Research, 16, 388–397. 10.4209/aaqr.2014.11.0292

[gh2178-bib-0054] Zhou, Z. , Dionisio, K. L. , Arku, R. E. , Quaye, A. , Hughes, A. F. , Vallarino, J. , Spengler, J. D. , Hill, A. , Agyei‐Mensah, S. , & Ezzati, M. (2011). Household and community poverty, biomass use, and air pollution in Accra, Ghana. PNAS, 108(27), 11,028–11,033. 10.1073/pnas.1019183108 PMC3131368 21690396

