# Supplementary material for: Air Quality Impacts at an E‐Waste Site in Ghana Using Flexible, Moderate‐Cost and Quality‐Assured Measurements
Source: Geohealth. 2020 Aug 1;4(8):e2020GH000247. doi: 10.1029/2020GH000247 (PMC7431652; doi:10.1029/2020GH000247)
Supplement: Supplementary file 1 — Supporting Information S1 [file GH2-4-e2020GH000247-s001.pdf]

Air Quality Impacts at an E-Waste Site in Ghana using Flexible, Low-Cost and Quality Assured Measurements

Lawrencia Kwarteng,[1] Emmanuel Acquah Baiden,[1] Julius Fobil,[1] Stuart Batterman[2,*]

SUPPLEMENTAL INFORMATION

June 19, 2020

Figure S1. E-waste monitoring site 2. Top left: Site detail. Top right: Nearby area viewing south to burn location. C. Map detail around monitoring site 2. 2
Figure S2. Upwind monitoring site 1. Top left: Site location. Top right: View looking north towards e-waste site. Bottom: Map detail. Temporary waste/dredge heaps shown as cross-hatched pink area. 3
Figure S3. Downwind monitoring site. Top left: Site location. Top right: View towards southwest towards e-waste site. Bottom: Map detail. 4
Figure S4. Photo of sampling cage with monitoring instruments mounted on building at site 3. 5
Figure S5. Detail of impaction disk on $PM_{2.5}$ sampler showing excess deposition of particulate matter. The accumulation of PM formed conical projections (~4 mm high) on the sampler's oiled impaction disk directly below each of the sampler's seven jets. 5
Figure S6. Daily precipitation recorded at the Kotoka Airport and the upwind monitoring site for 2017-2018. . 6
Figure S7. Monthly precipitation recorded at the Kotoka Airport and at the Upwind Monitoring Site for 2017-2018. Long term average from https://www.weather-atlas.com/en/ghana/accra-climate#rainfall. 6
Figure S8. Wind roses at by time of day. Based on 2017 – 2018 data at Kotoka airport. 7
Figure S9. Wind roses at by season. Based on 2017 – 2018 data at Kotoka airport. 7
Figure S10. Comparison of gravimetric and optical $PM_{2.5}$ concentrations. Left: 24-hr samples at upwind, e-waste and downwind sites. Right: 4-hr samples for e-waste workers 8
Figure S11. PM concentrations by hour of day for two study periods. 8
Figure S12. Daily aerosol optical depths over region for month of November 2017. Location of Accra indicated with star. Each map shows a 1450 x 1450 km region. 13
Figure S13. Daily aerosol optical depths over region for month of December. 14
Figure S14. Daily aerosol optical depths over region for month of January 2018. 15
Figure S15. Daily aerosol optical depths over region for month of February 2018. 16
Figure S16. Photo of EPA Graphic Road PM monitor in cage in middle of street. From Google Street View. 17
Figure S17. PM_{10} concentrations in 2017 for the non-Harmattan period at the EPA monitors (permanent monitors in blue; roadside monitors in green) in Accra, and at the upwind, e-waste and downwind monitors in this study (red). 17

Table S1. Statistics of 1-hr optical PM data.
Table S2. Chronology interpreting satellite record of AOD over Ghanat from November 2017 to February 2018.
Table S3. Summary of ambient monitoring data reported by the Ghanaian Environmental Protection Agency for 2015
Table S4. Summary of ambient monitoring data reported by the Ghanaian Environmental Protection Agency for 2016
Table S5. Summary of ambient monitoring data reported by the Ghanaian Environmental Protection Agency for 2017

Figure S1. E-waste monitoring site 2. Top left: Site detail. Top right: Nearby area viewing south to burn location. C. Map detail around monitoring site 2.

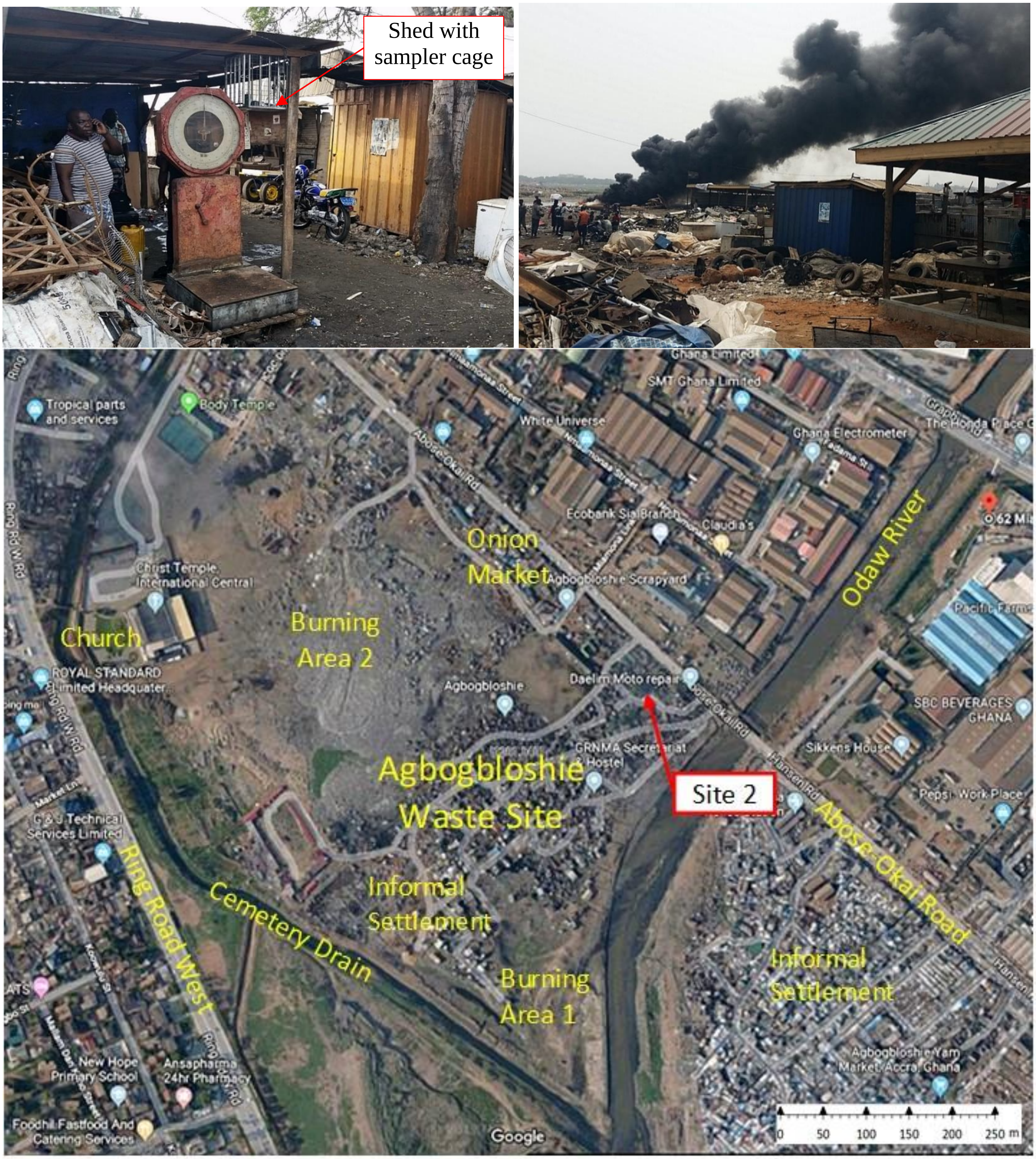

Figure S2. Upwind monitoring site 1. Top left: Site location. Top right: View looking north towards e-waste site. Bottom: Map detail. Temporary waste/dredge heaps shown as cross-hatched pink area.

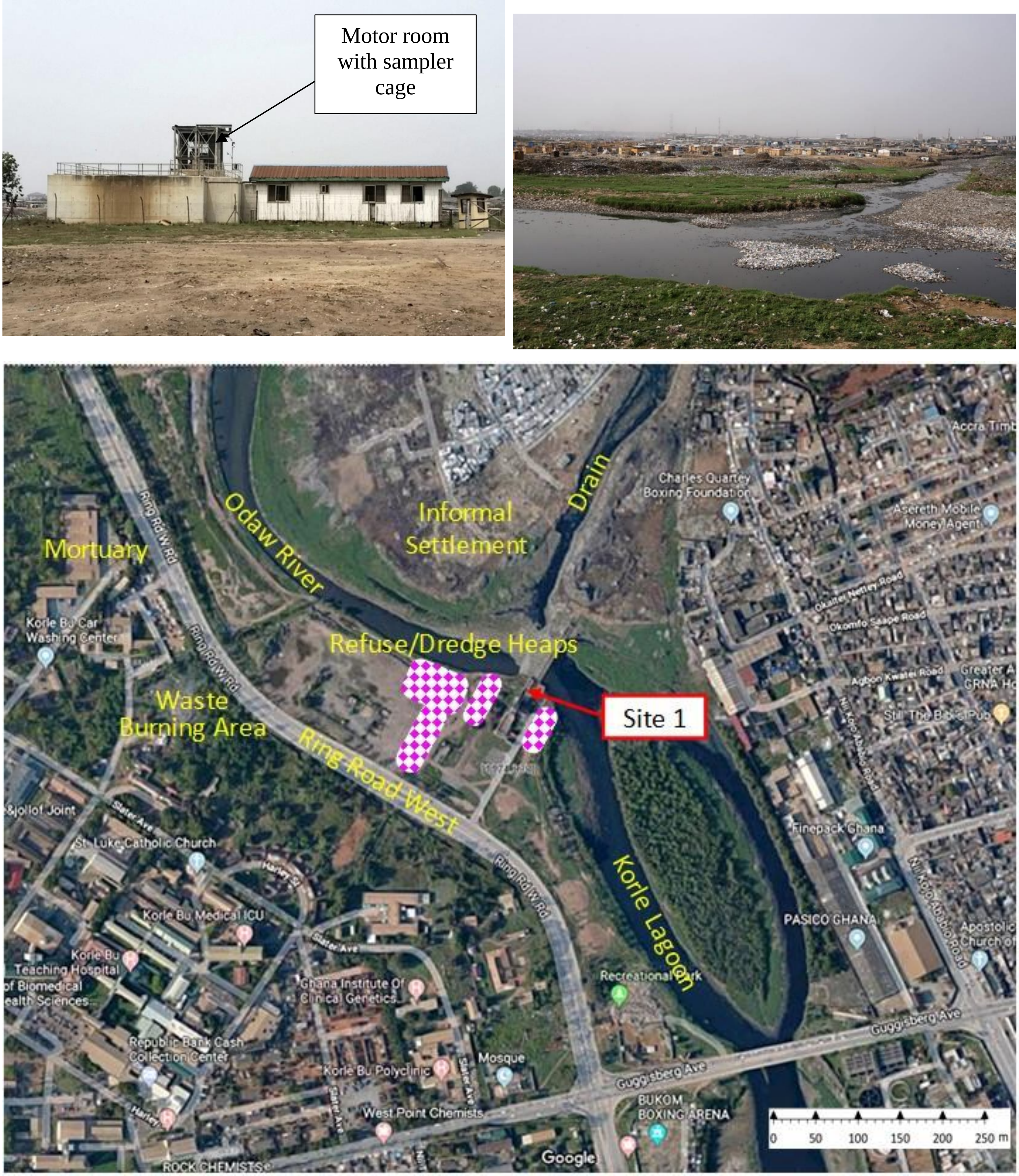

Figure S3. Downwind monitoring site. Top left: Site location. Top right: View towards southwest towards e-waste site. Bottom: Map detail.

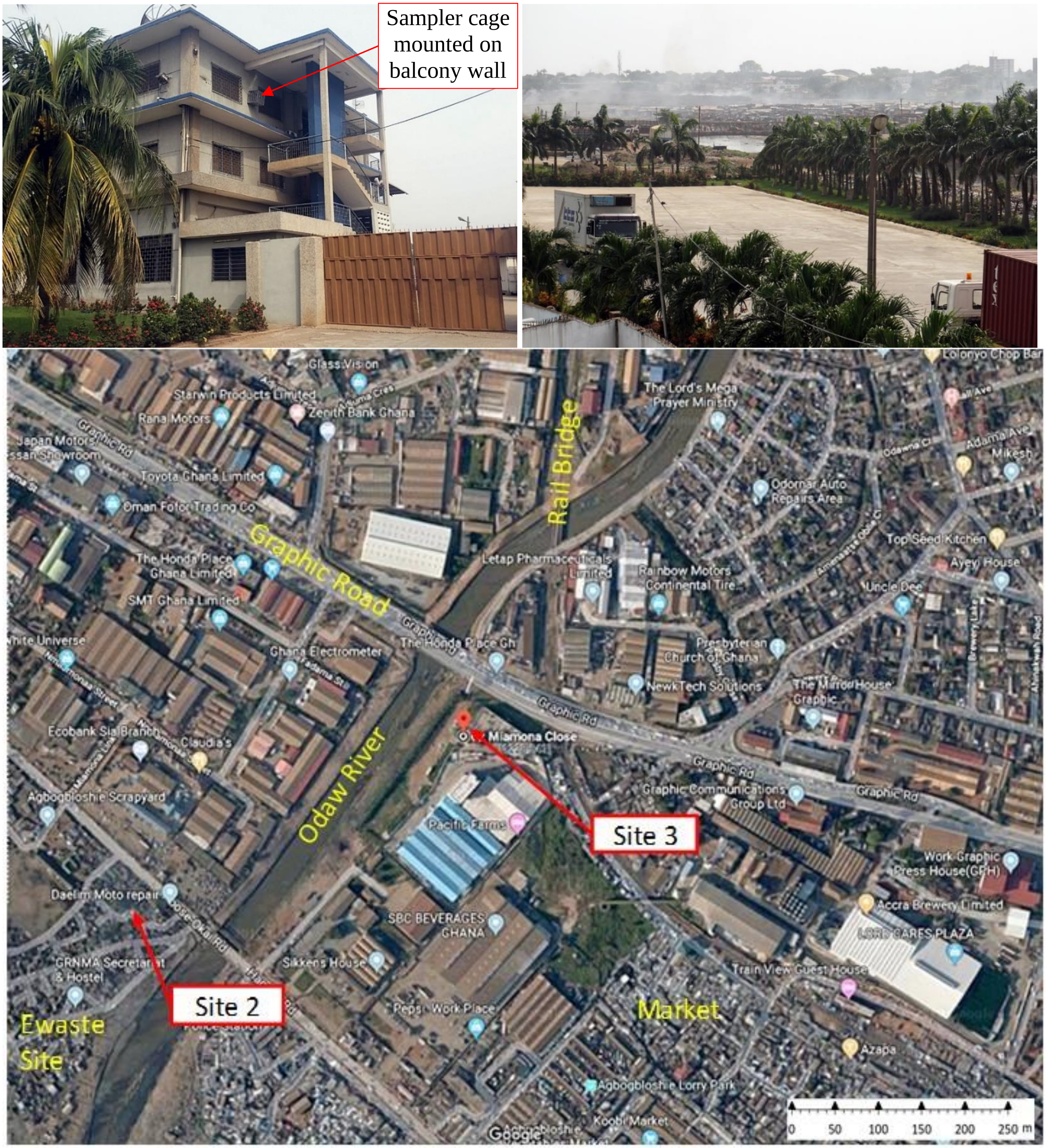

Figure S4. Photo of sampling cage with monitoring instruments mounted on building at site 3.

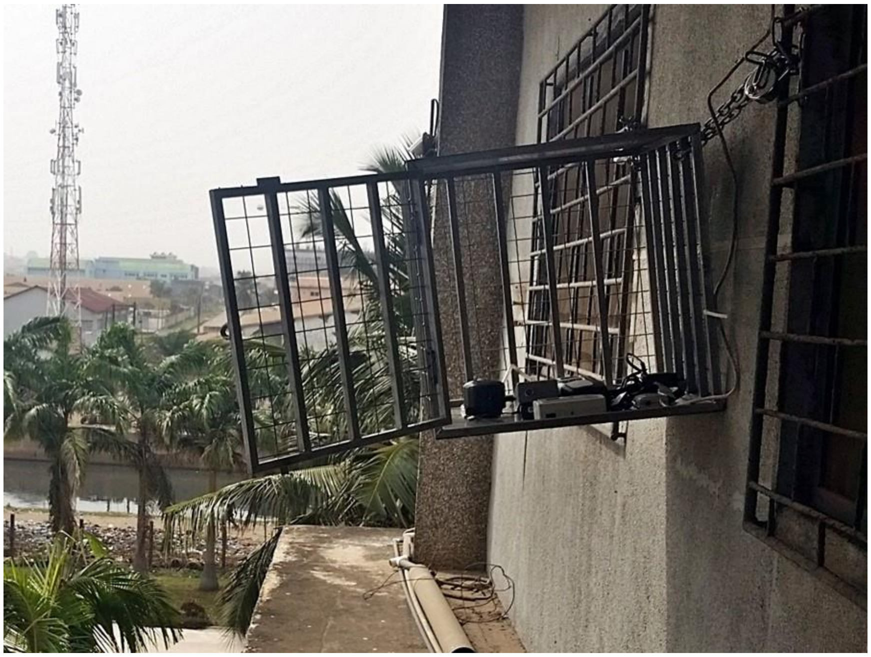

Figure S5. Detail of impaction disk on $PM_{2.5}$ sampler showing excess deposition of particulate matter. The accumulation of PM formed conical projections (~4 mm high) on the sampler's oiled impaction disk directly below each of the sampler's seven jets.

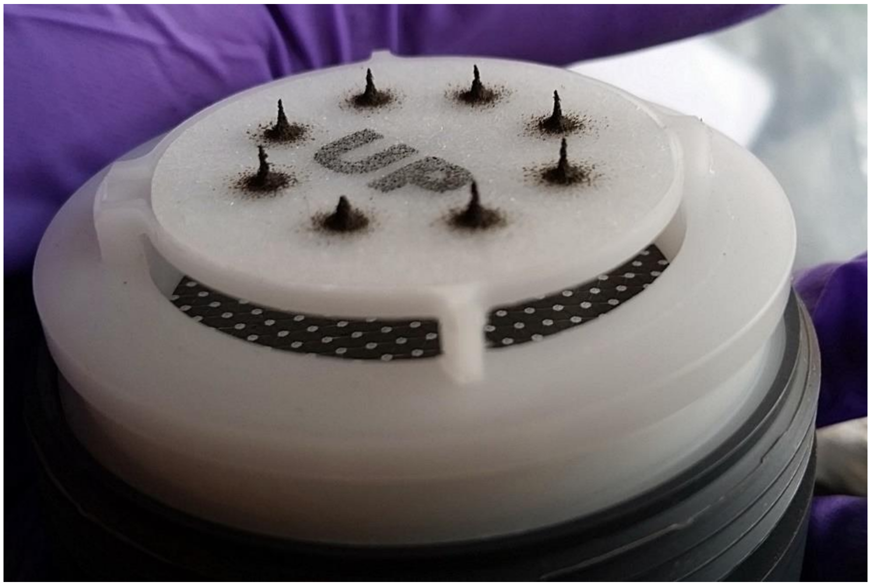

1 Comparison of precipitation at the two sites

Precipitation data at the Kotoka Airport and upwind monitoring site was compared at hourly, daily and monthly levels. The figures below show daily and monthly trends. The two sites showed similar trends, although precipitation quantities and times varied, i.e., precipitation often fell on different hours during the day. The data record at the upwind site is available from 7/30/17 to 8/28/18. Observation appear to be missing from the Kotoka Airport data, and the data treatment was not consistent, leading to some values apparently being repeated on a second hour.

June and May have the most rainfall (178 – 142 mm), followed by April, October and March; August and January are the driest months (15 mm). Based on the Kotoka Airport data, measurable precipitation occurred on an average of 74 days per year; considering daily precipitation over 5 mm, on 30 days per year. The recorded precipitation total at Kotoka Airport averaged 882 mm, which exceeds the long-term average of 725 mm. The short rainfall events and gaps in the record did not allow good concordance with the PM data.

Figure S6. Daily precipitation recorded at the Kotoka Airport and the upwind monitoring site for 2017-2018.

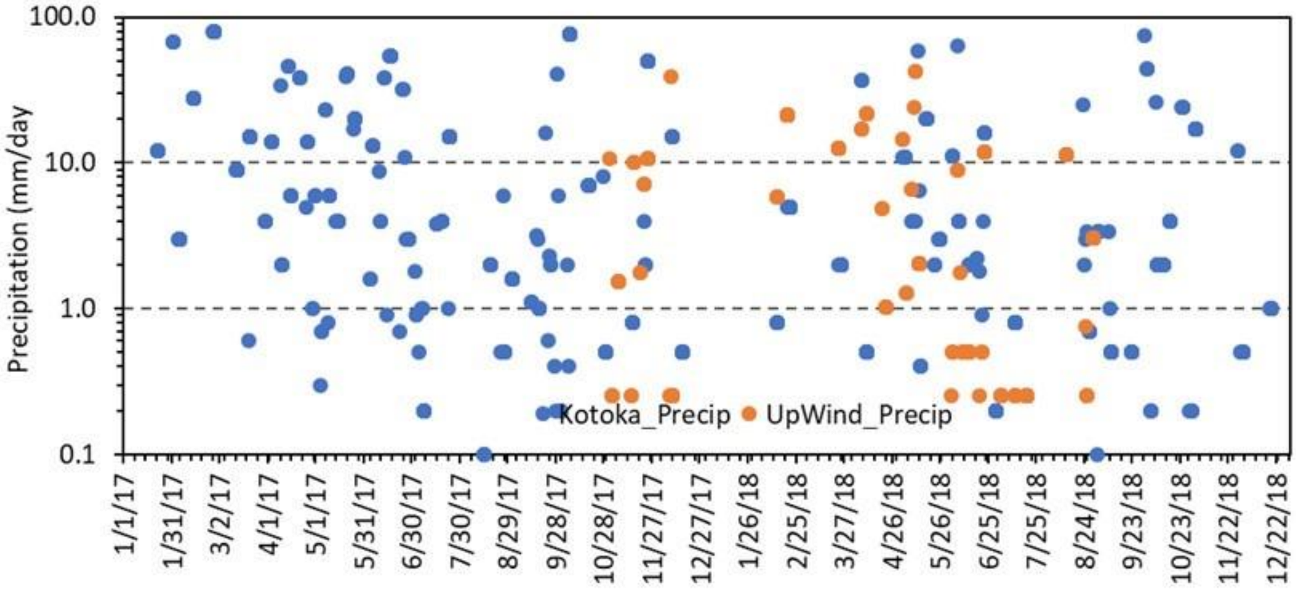

Figure S7. Monthly precipitation recorded at the Kotoka Airport and at the Upwind Monitoring Site for 2017-2018. Long term average from https://www.weather-atlas.com/en/ghana/accra-climate#rainfall.

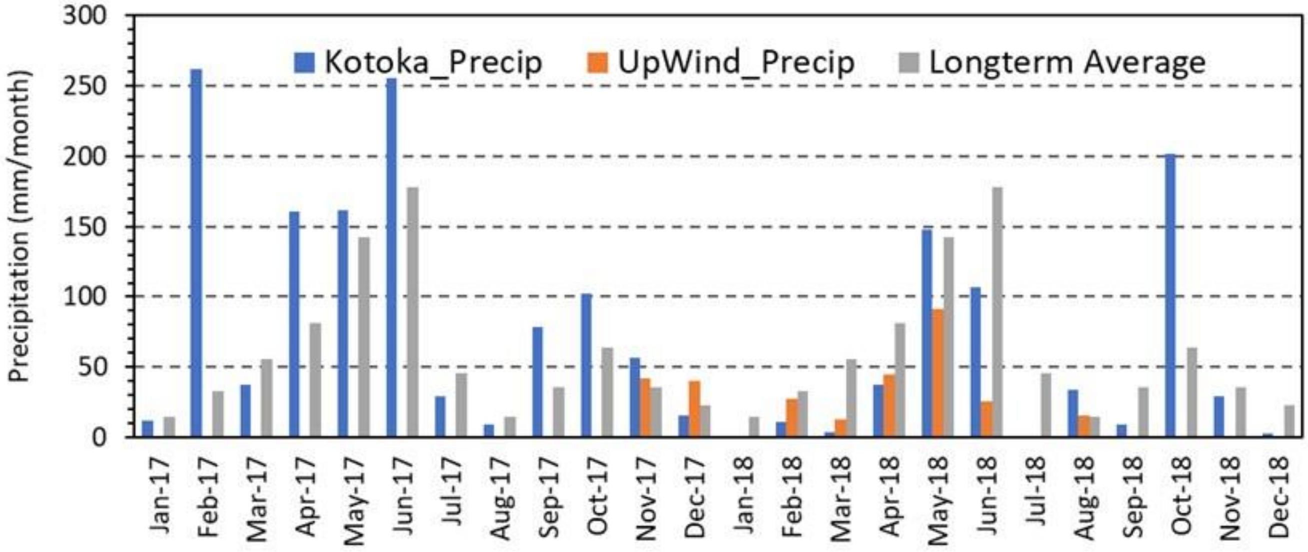

Figure S8. Wind roses at by time of day. Based on 2017 – 2018 data at Kotoka airport.

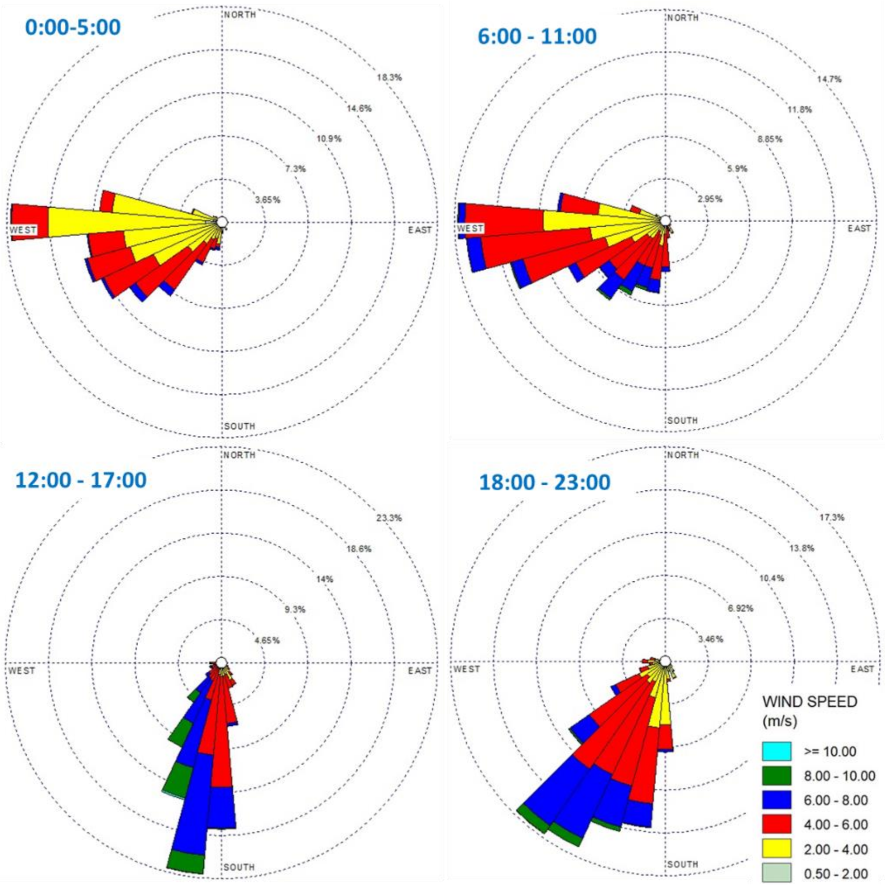

Figure S9. Wind roses at by season. Based on 2017 – 2018 data at Kotoka airport.

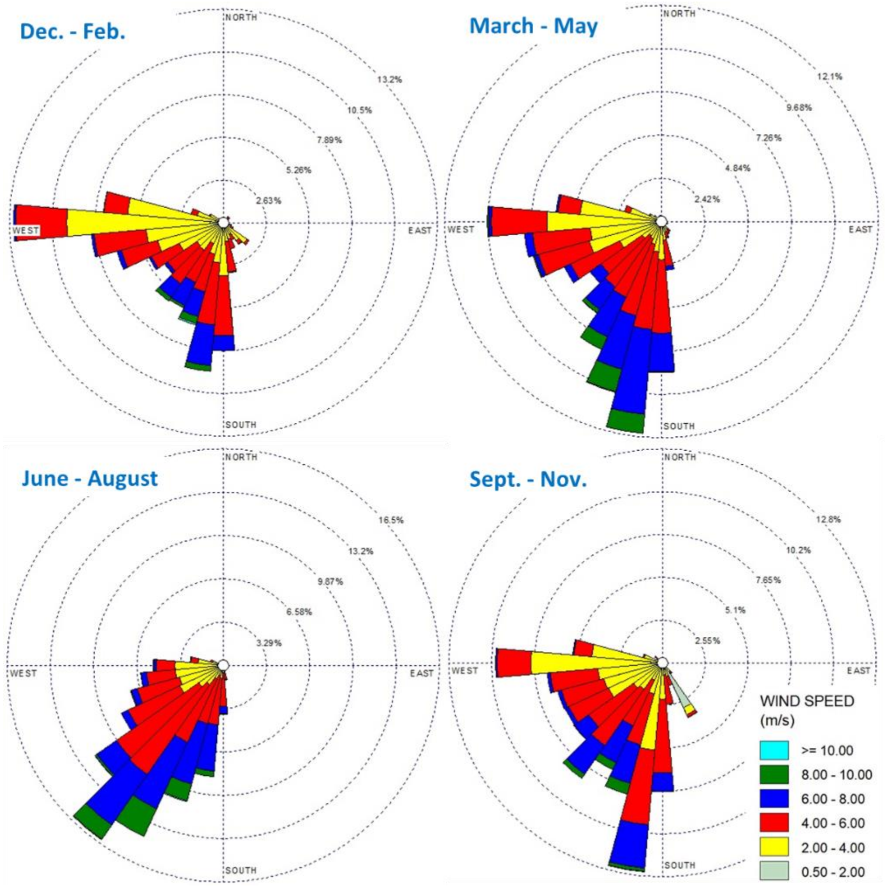

Figure S10. Comparison of gravimetric and optical $PM_{2.5}$ concentrations. Left: 24-hr samples at upwind, e-waste and downwind sites. Right: 4-hr samples for e-waste workers

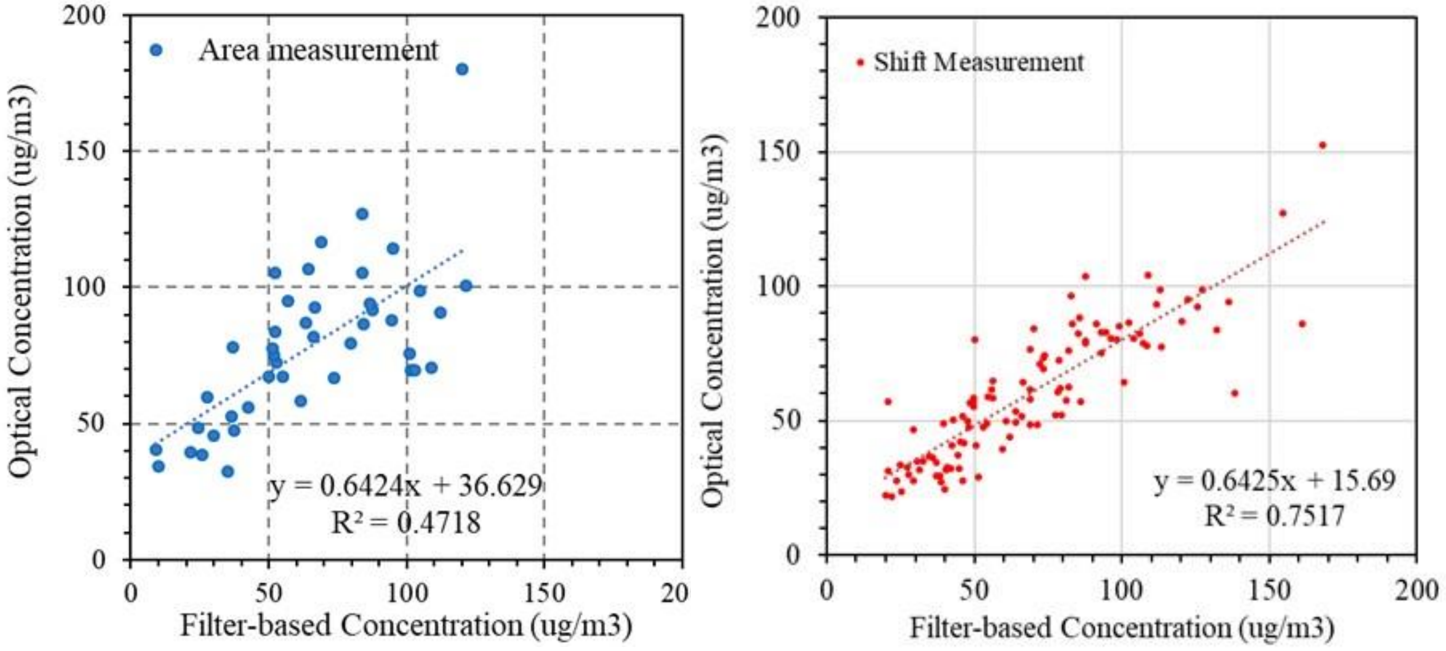

Figure S11. PM concentrations by hour of day for two study periods.

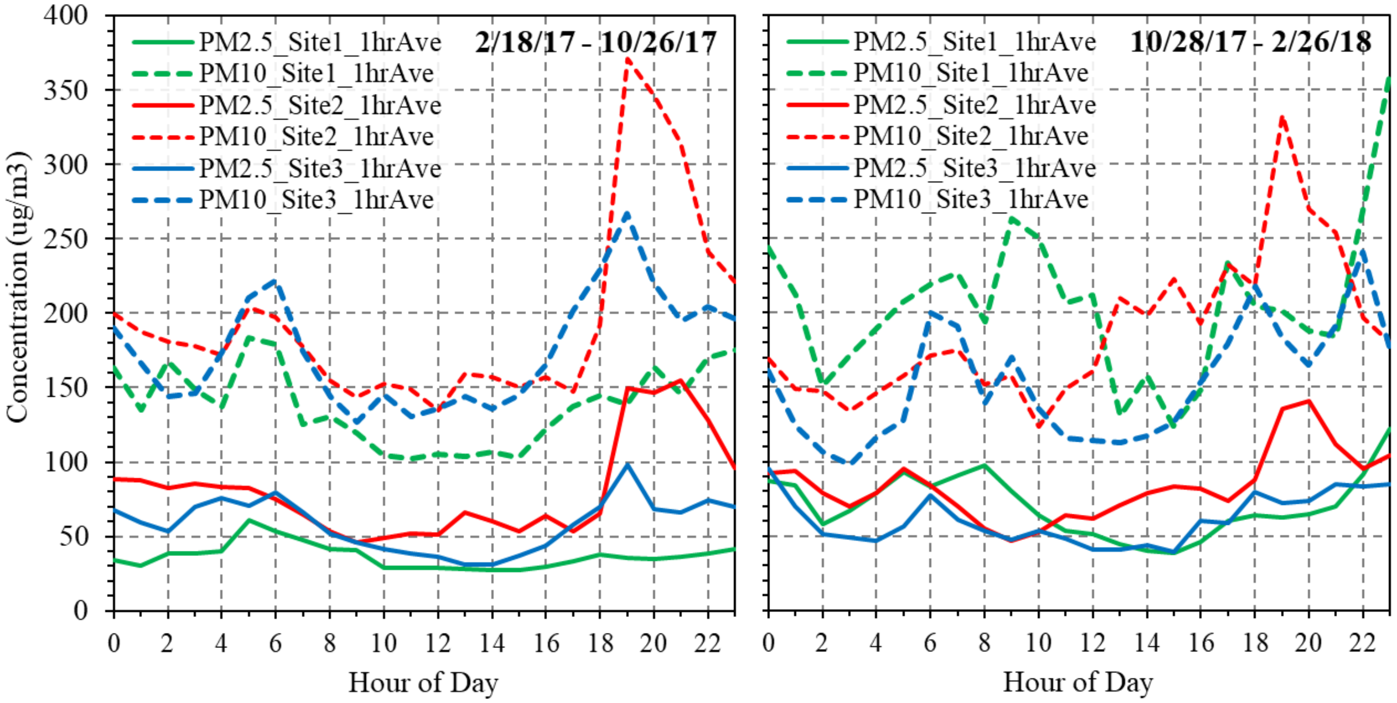

Table S1. Statistics of 1-hr optical PM data for three periods (entire study, non-Harmattan season, and Harmattan season).

PM	Sample	2/18/17 to 2/26/18				2/18/17 to 10/27/17				10/28/17 - 2/26/18			
Size	Type	Site1	Site2	Site3	All	Site1	Site2	Site3	All	Site1	Site2	Site3	All
**PM2.5**	Average	71	90	67	78	44	88	68	74	92	93	66	86
	Median	52	75	55	64	38	72	54	60	68	78	58	70
	St. Dev.	72	58	42	58	23	58	44	52	88	56	36	67
	Min	7	8	11	7	7	13	11	7	12	8	20	8
	5th Percentile	19	28	23	22	18	29	23	22	23	21	25	23
	25th Percentile	33	52	38	41	28	51	37	40	43	60	41	46
	75th Percentile	82	110	84	94	56	108	83	91	94	112	85	99
	95th Percentile	209	205	150	191	91	205	154	183	297	199	142	207
	Max	523	515	279	523	135	515	279	515	523	380	206	523
	NOBs	571	1040	707	2318	248	729	484	1461	323	311	223	857
**PM10**	Average	216	215	188	207	159	212	190	196	260	221	186	227
	Median	169	183	163	175	137	181	167	171	197	195	152	184
	St. Dev.	160	133	103	133	80	127	98	113	190	145	115	160
	Min	36	15	39	15	36	31	39	31	49	15	41	15
	5th Percentile	67	70	62	67	61	81	66	71	73	33	59	60
	25th Percentile	116	130	117	123	102	130	124	123	133	129	102	122
	75th Percentile	271	260	235	254	196	253	235	240	332	279	242	290
	95th Percentile	501	499	392	454	323	478	385	412	625	535	418	531
	Max	1281	865	684	1281	427	865	622	865	1281	825	684	1281
	NOBs	571	1040	707	2318	248	729	484	1461	323	311	223	857

Satellite observations

To investigate potential of dust loadings due to Harmattan winds, we utilized two products from the Moderate-Resolution Imaging Spectroradiometer (MODIS) satellites that provide daily estimates. The first uses the aerosol optical depth (AOD) based on the MODIS dark target algorithms and daytime observations from Terra and Aqua satellites. The AOD estimates have 10 km resolution (at best); the visual background is resolved to 2 km. We extracted daily maps for spanning Nov. 2017 to Feb. 2018 in Figures S12-S15. On the maps, the location Accra is indicated by a yellow star. The second estimate uses the Combined Value-Added Aerosol Optical Depth layer (CVA-AOD), provided on ~56 km grid, which is designed for quantitative applications including aerosol data assimilation and model validation. The CVA-AOD layer has more consistent performance over brighter targets but less spatial resolution.

The interpretation of AOD with respect to Harmattan dusts and PM levels is not direct. Overall, AODs below 0.1 indicate clear skies with maximum visibility, while AODs above 1 indicates the presence of dense aerosols with difficulty seeing the Sun. The maps represent AODs based on two instantaneous readings, and are not a daily average. Daily coverage is incomplete at the equator, and locations that are not covered are indicated by black bands on the maps. The usual MODIS resolution of 10 km is much coarser at the edge of a swath. The AOD algorithms vary over land and sea, and a dark background, e.g., vegetation, is needed to obtain consistent results, and AOD is not determined over light backgrounds, e.g., ocean glints and desert. AOD is integrated over the height of the atmosphere, and may not reflect PM levels near the ground for many reasons. The land-sea interface complicates both the AOD determinations and wind patterns, and often Accra is on the edge of a band, possibly due to the consistent S and SW winds. With these caveats, we can suggest a general patterns that may indicate Harmattan dusts: higher values of AOD (>1 or 2) that are more or less continuous (indicated by larger red areas on the maps), particularly if they are moving from the NE.

Link to AOP data:
https://worldview.earthdata.nasa.gov/?v=-37.84531304206394,-6.14357980400378,26.807030707936057,26.481420195996222&t=2018-03-12-T00%3A00%3A00Z&as=2017-09-05-T00%3A00%3A00Z&ae=2018-03-15-T00%3A00%3A00Z&l=MODIS_Aqua_Aerosol_Optical_Depth_3km,Reference_Labels,Reference_Features,Coastlines(hidden),VIIRS_SNPP_CorrectedReflectance_TrueColor(hidden),MODIS_Aqua_CorrectedReflectance_TrueColor,MODIS_Terra_CorrectedReflectance_TrueColor(hidden)&av=3.5&ab=on

Link to CVA-AOP data:
https://worldview.earthdata.nasa.gov/?v=-39.0172899528242,-7.173971401958093,28.482710047175797,26.57602859804191&t=2018-03-28-T00%3A00%3A00Z&as=2017-09-01-T00%3A00%3A00Z&ae=2017-09-30-T00%3A00%3A00Z&l=MODIS_Combined_Value_Added_AOD(opacity=0.69),MODIS_Aqua_Aerosol_Optical_Depth_3km(hidden,opacity=0.69),Reference_Labels,Reference_Features,Coastlines(hidden),VIIRS_SNPP_CorrectedReflectance_TrueColor(hidden),MODIS_Aqua_CorrectedReflectance_TrueColor,MODIS_Terra_CorrectedReflectance_TrueColor(hidden)&av=0.5&ab=on

Table S2. Chronology interpreting satellite record of AOD over Ghanat from November 2017 to February 2018.

November, 2017. The month is clear with several exceptions, but these appear unlikely to represent Harmattan dusts. The AOD and combined products are largely consistent.

CVA-AOD results:

Nov 1	Coastal band moved W leaving Accra and coast clear. Patches of AOD N of Accra .
Nov 3-5	AOD≈0.3 in patchy coastal band, but Accra remains clear.
Nov 6	Clear skies over Accra and coast.
Nov 7	Dense band (AOD≈0.6) moves W throughout eastern Ghana. Patches above Accra.
Nov 8–9	Clear skies over Accra and the coast.
Nov 10-14	AOD≈0.4. Coastal band covers entire coast of country and some parts of Accra.
Nov 15-18	Clear skies over Accra and the coast.
Nov 19-21	AOD ≈ 0.2. Patch coastal band covering entire length of country's coast.
Nov 20-25	Clear skies over Accra and the coast.
Nov 26–27	AOD≈0.3 moves W along coast and over portions of Accra.
Nov 28 – 30	AOD≈ 0.4. Coastal band moves from west to east along the coast.

AOD results: The higher AOD periods near Accra are described below and shown on Fig. S12.

Nov. 8-15:	Large band with AOD ≈ 3 that is inland by 50 km, but does not reach Accra
Nov. 26-30	Broken coverage with AOD ≈ 1, but again, the band generally does not reach Accra
Nov. 28	Patch with AOD ≈ 3 that reaches Accra, but is short lived.

December 2017: The combined product shows moderate to high but patchy AOD over Accra from Dec. 10-16 and Dec. 24-31, suggesting Harmattan dusts at the end of the month.

CVA-AOD results:

Dec 1-7	Accra sandwiched between two bands moving W, but city remains clear.
Dec 8-9	Coastal patches, but Accra remains clear.
Dec 10-16	Dense patches (AOD≈5) moving W over entire coastal area including Accra.
Dec 17,19-23	Clear skies over Accra.
Dec 18	Dense coastal band. No data for Accra.
Dec 24-31	Dense band (AOD≈5) covering all of southern Ghana. (Dec 27 has no data).

AOD results: The higher AOD periods near Accra are described below and shown in Fig. S13:

Dec. 1-6	Fairly consistent near-coastal band with AOD ≈ 1 continues
Dec. 12-18	Patchy areas with AOD ≈ 3 but these appear just north of Accra.
Dec. 24	Wide band with spots reaching AOD ≈ 4 largely SW of Accra, but clear skies to the NE.
Dec. 26-30	Band just inland with AOD ≈ 3; this appears to moving SW.

January 2018: Several periods with potentially widespread Harmattan dusts are indicated by moderate to high AOD over Accra on Jan. 1-6, possibly on 8-14, and on 16-18, 22-24, 27-28, and 31. However, clear skies are indicated for Jan. 7, 25, 28 and 30.

CVA-AOD results:

Jan 1-6	Dense band (AOD≈5) moves E over Accra.
Jan 7	Band north of Accra but the city is clear.
Jan 8-24	Dense band (AOD≈0.5-5) moves E over Accra; some days are patchy over Accra. No data for Jan 12, 19 and 21.
Jan 26	Band E of Accra
Jan 27-29	Dense band (AOD≈5) over Accra and entire southern Ghana
Jan 29	Patches cover Accra and form coast.
Jan 30	Coastal band close to Accra.
Jan 31	Large band over entire country

AOD results: The higher AOD periods near Accra are described below and shown in Fig. S14.

Jan. 1-6	Narrow coastal band with AOD ≈ 3.
Jan. 17-19	Narrow but discontinuous band around Accra with AOD ≈ 2.
Jan 22-24	Patchy areas around Accra with AOD ≈ 1
Jan. 27	Coastal bad with AOD ≈ 2

February 2018: Generally, AOD maps show patches over Accra with moderate AOD on Feb. 1-2, 7-8, but generally few if any periods of widespread Harmattan dusts are indicated.

CVA-AOD results:

Feb 1	Dense band (AOD≈5) over Accra, continued from January
Feb 2	Patches N and S of Accra
Feb 4-5	Large dense band N and E, but Accra remains clear.
Feb 7-8, 10	Moderately dense band (AOD≈0.6) to N, patches to S, Accra covered most days
Feb 11	Clear skies
Feb 12-14	Moderately high band (AOD≈0.6) over Accra.
Feb 15-18	Clear skies
Feb 19-21	Patches of coastal band, but not over Accra
Feb 22-27	Clear skies above Accra .
Feb 28	Light patches (AOD≈0.1) N and E of Accra

AOD results:

Feb. 5-8	Patches around Accra with AOD ≈ 1
Feb. 10	Patches around Accra with AOD ≈ 1
Feb. 19	Patches around Accra with AOD ≈ 1
Feb. 22	Patches around Accra with AOD ≈ 1

Figure S12. Daily aerosol optical depths over region for month of November 2017. Location of Accra indicated with star. Each map shows a 1450 x 1450 km region.

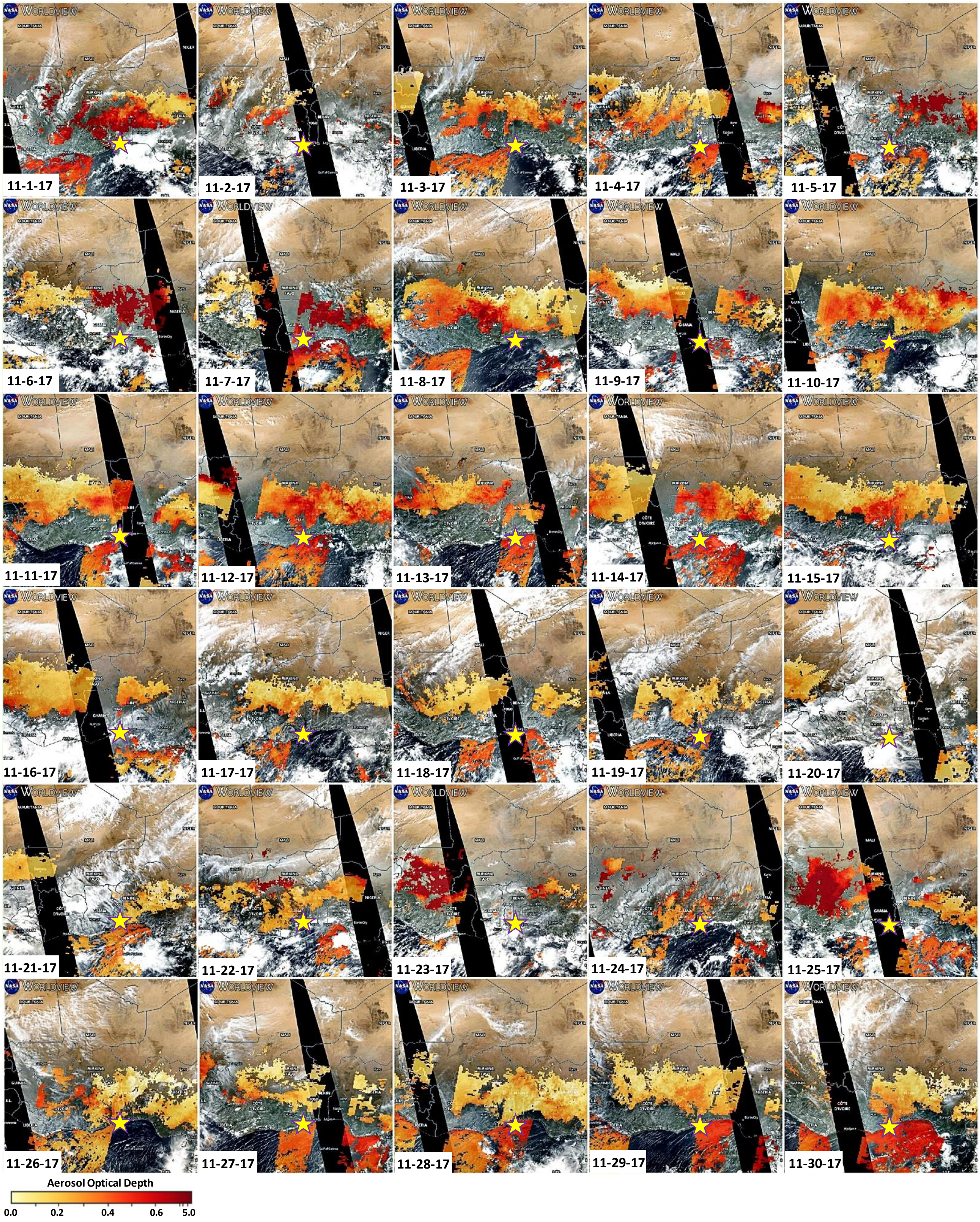

Figure S13. Daily aerosol optical depths over region for month of December.

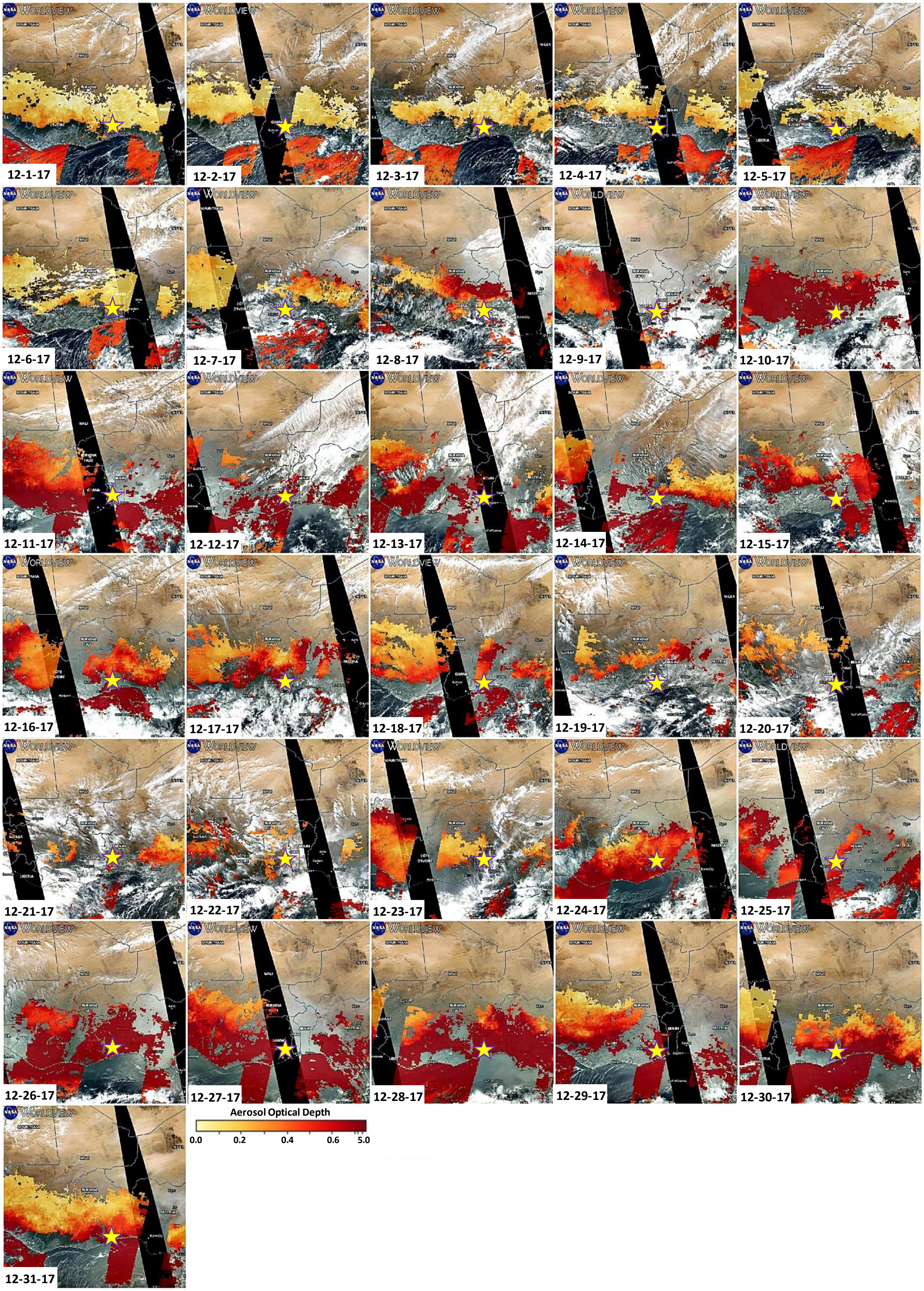

Figure S14. Daily aerosol optical depths over region for month of January 2018.

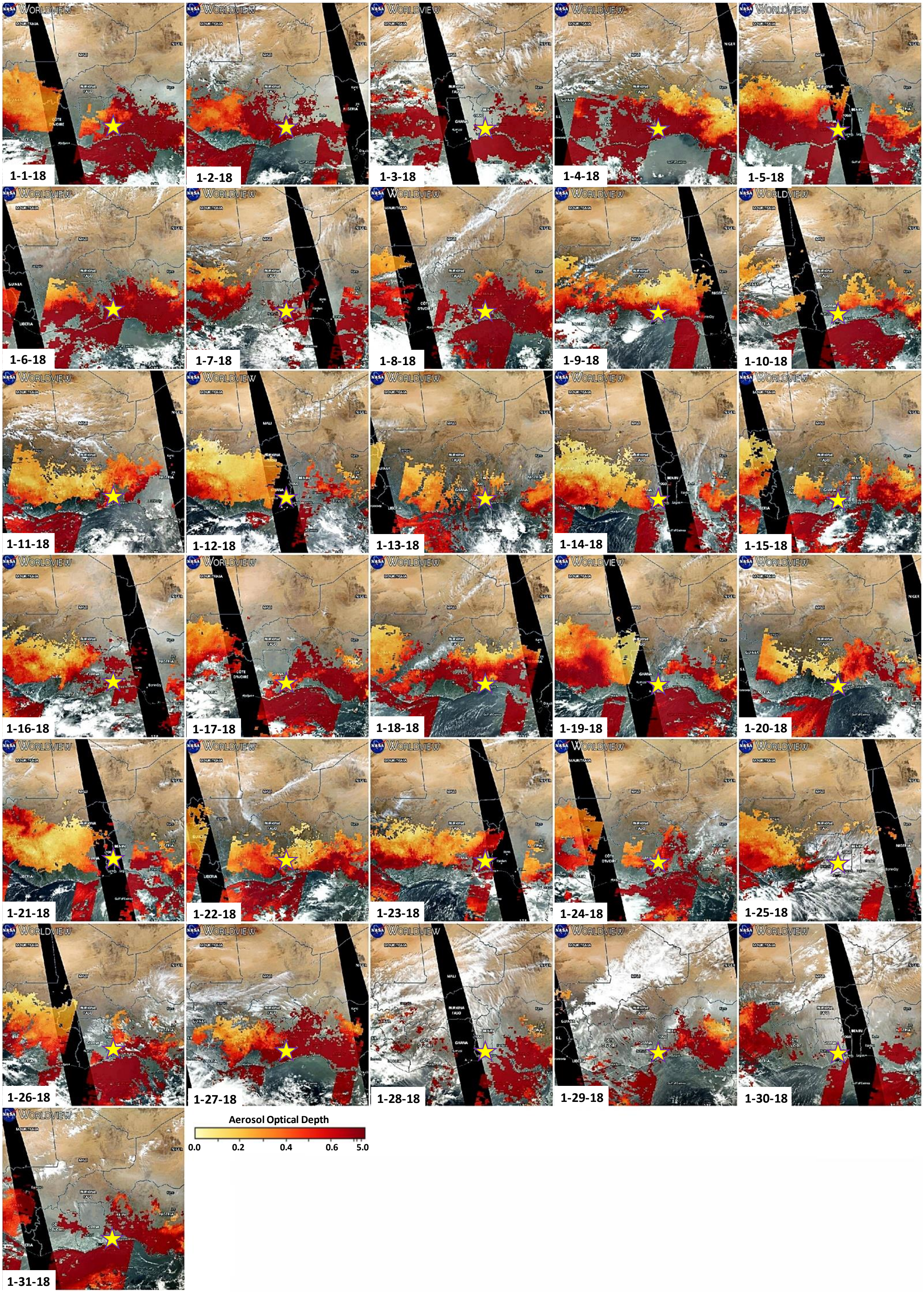

Figure S15. Daily aerosol optical depths over region for month of February 2018.

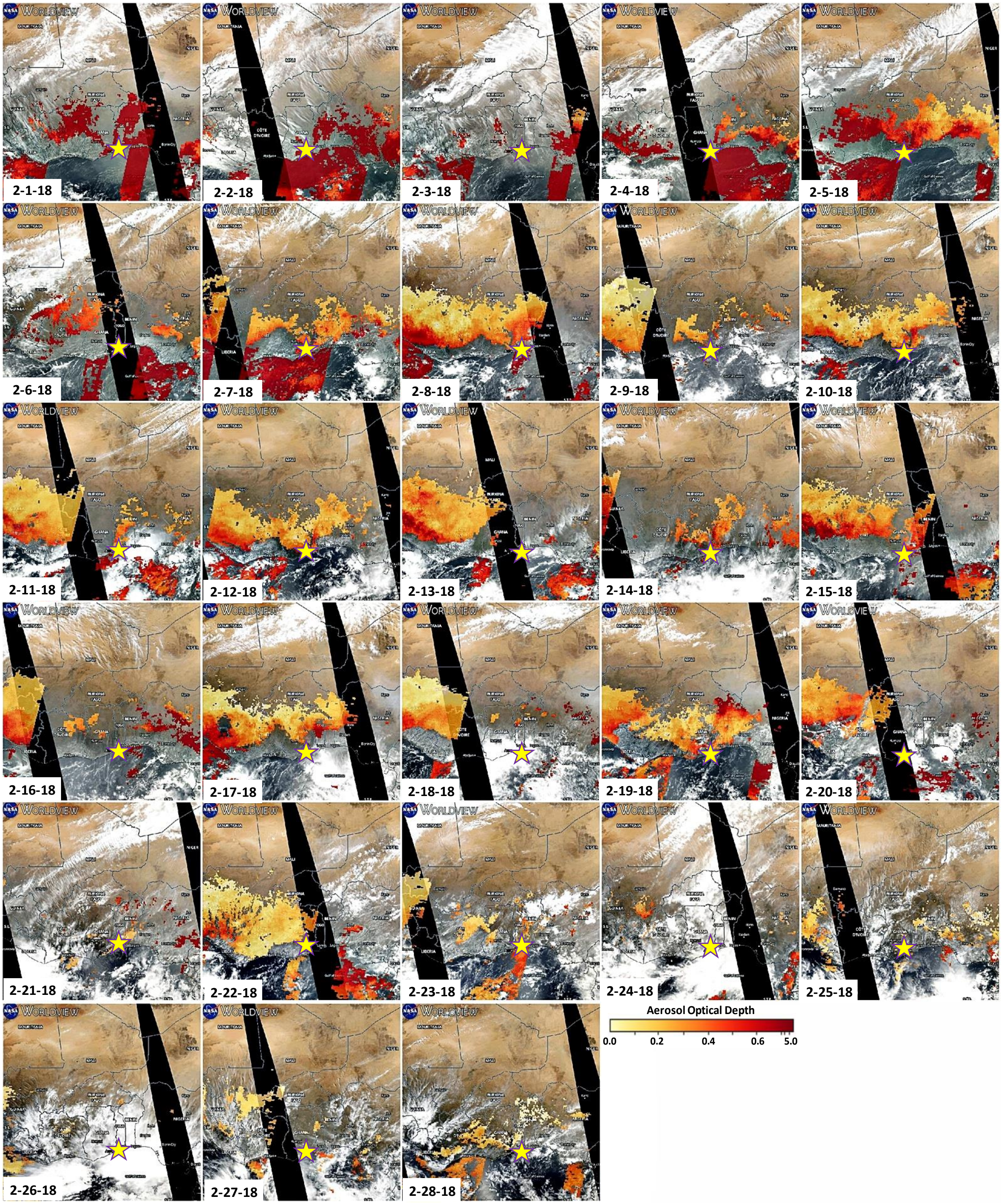

Figure S16. Photo of EPA Graphic Road PM monitor in cage in middle of street. From Google Street View.

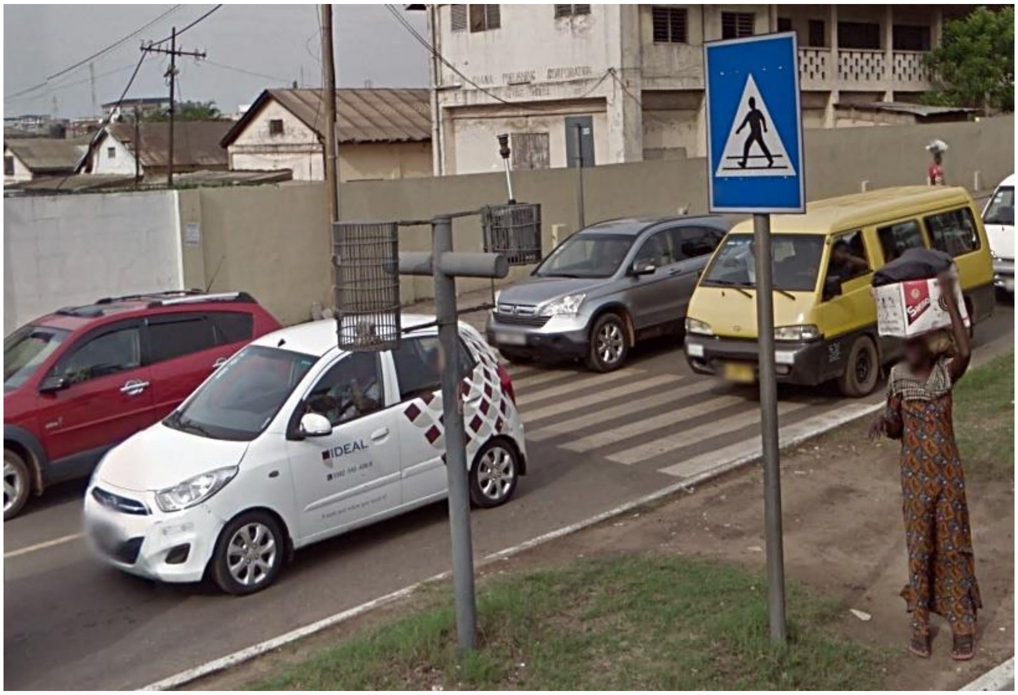

Figure S17. PM_{10} concentrations in 2017 for the non-Harmattan period at the EPA monitors (permanent monitors in blue; roadside monitors in green) in Accra, and at the upwind, e-waste and downwind monitors in this study (red).

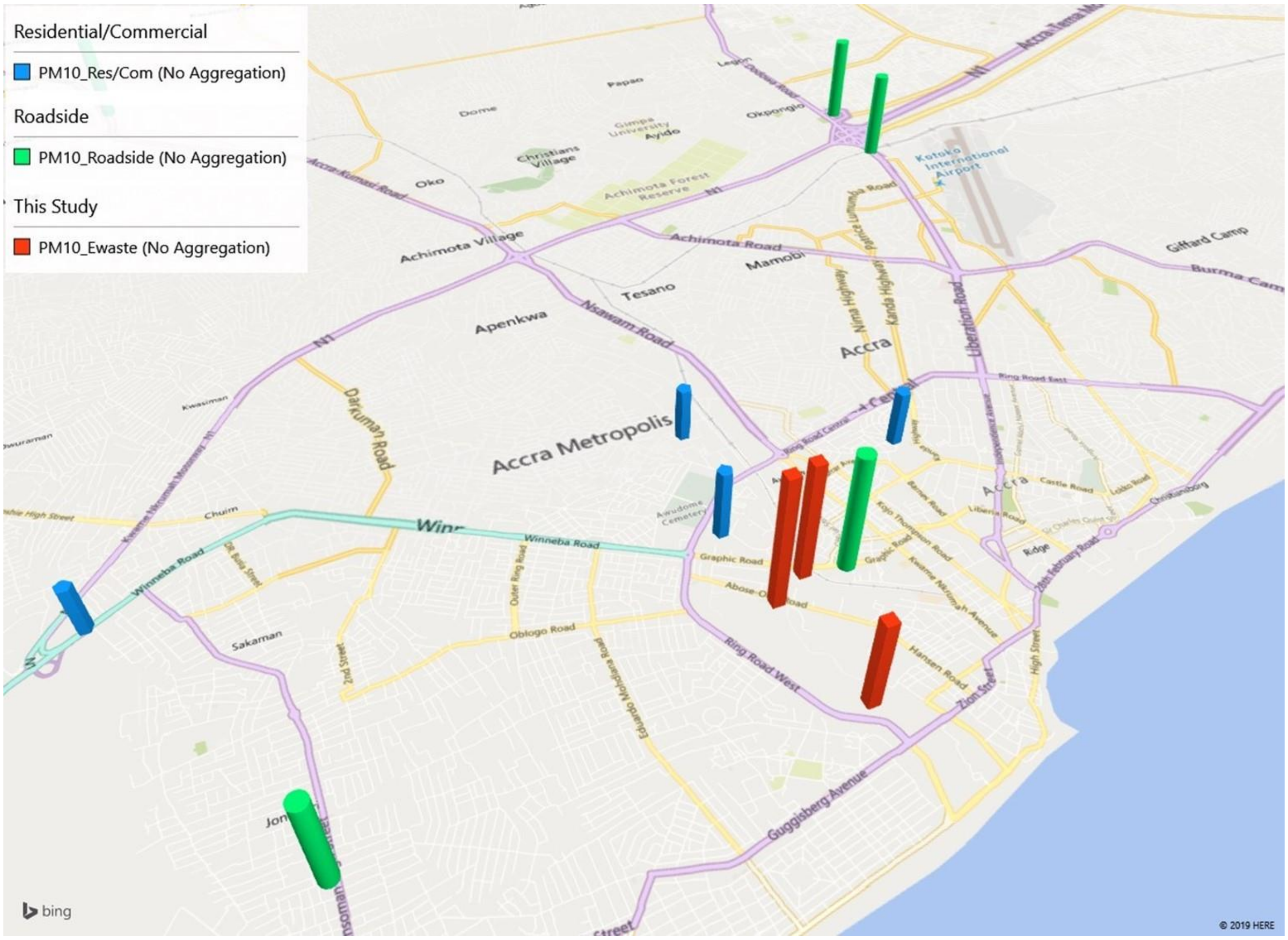

Table S3. Summary of ambient monitoring data reported by the Ghanaian Environmental Protection Agency for 2015 for sites with sufficient data to calculate seasonal averages. Concentrations in µg/m^3. See below for sample size and for additional roadside sites.

	Permanent - PM2.5					Permanent - PM10					Roadside PM2.5					Roadside PM10						
Month or Period	Danosoman	North_Industrial_Area	South_Industrial_Area	Odorkor	4-site Average	Danosoman	North_Industrial_Area	South_Industrial_Area	Odorkor	4-site Average	Graphic_Road	Mallam_Market	Weija_Junction	Shangri_La	4 -site Average	Graphic_Road	Mallam_Market	Weija_Junction	Kasoa	Shangri_La	Achimota	6 -site Average
Jan	-	-	-	-	-	255	111	112	298	194	-	-	-	-	-	222	635	337	264	591	561	435
Feb	-	-	-	-	-	48	124	58	116	87	-	-	-	-	-	146	94	306	174	115	84	153
Mar	-	-	-	-	-	-	83	102	-	93	94	60	157	103	104	168	146	224	146	129	121	156
Apr	-	-	-	-	-	-	94	109	230	144	97	176	176	208	164	194	112	410	224	135	112	198
May	69	35	97	63	66	-	74	75	6	52	120	111	243	125	150	140	84	297	143	121	70	143
Jun	-	21	-	201	111	-	78	74	-	76	76	49	-	35	53	256	109	298	136	70	49	153
Jul	-	-		146	146	100	53	45	68	67	98	56	97	56	77	56	59	81	146	59	67	78
Aug	21	37	56	-	38	114	111	63	167	114	-	62	104	-	83	118	90	135	146	62	101	109
Sep	28	69	42	90	57	104	98	94	107	101	69	62	153	59	86	60	79	126	136	56	56	86
Oct	65	116	83	157	105	148	81	60	42	83	98	104	-	132	111	180	115	171	159	84	84	132
Nov	56	112	132	104	101	-	-	81	-	81	-	-	-	-	-	65	56	295	-	260	260	187
Dec	209	-	-	208	209	160	129	100	94	121	103	-	140	-	122	148	279	262	-	144	267	220
Average by month	75	65	82	138	104	133	94	81	125	101	94	85	153	103	105	146	155	245	167	152	153	171
by sample	71	70	78	78	-	117	95	83	118	-	95	92	158	112	152	149	155	247	170	151	152	152
Average Mar-Nov	48	65	82	127	89	117	84	78	103	90	93	85	155	103	103	137	94	226	155	108	102	138
Dec-Feb	209	-	-	208	209	154	121	90	169	134	103	-	140	-	122	172	336	302	219	283	304	269

The permanent sites include both $PM_{2.5}$ and PM_{10} monitors. The sample size for the annual averages at these sites is 16-17 for $PM_{2.5}$ and 25-43 for PM_{10} measurements. Roadside monitoring at Graphic Road, Mallam Market, Weija Junction, Kason, Shangri La and Achimota had sample sizes from 44 to 55. In addition, EPA conducted monitoring at 4 other roadside sites (First Light, La Palma, Tantra Hill, Amasaman), but inadequate data was captured to construct meaningful averages. At the roadside sites, $PM_{2.5}$ comprised an average of 78% of PM_{10} in the non-Harmattan season, and 45% in the Harmattan season.

Table S4. Ghanaian EPA data summary for 2016.

Month or Period	East Legon	Dansoman	North Industrial Area	South Industrial Area	Odorkor	5-site Average	First light	Shangri La	Achimota	La palm	Mallam market	Graphic	Weija	Kasoa	Tantra Hill	Amasaman	11-site Average
	Permanent - PM10						Roadside PM10										
Jan	-	300	414	-	-	357	472	431	347	296	250	292	361	-	243	264	328
Feb	-	-	153	-	120	136	308	278	256	322	356	333	194	-	229	292	285
Mar	-	53.4	88	-	78	73	167	149	88	142	144	150	222	-	97	181	149
Apr	-	66.8	110	-	86.4	88	74	184	243	208	215	134	356	-	142	342	211
May	-	61.8	84.3	-	99.1	82	163	125	104	117	108	153	211	-	72	97	128
Jun	-	56.9	108	-	88	84	233	161	97	114	94	194	264	215	69	108	155
Jul	-	60	91	-	101	84	83	83	103	131	106			208	53	76	105
Aug	-	55	87	-	-	71	65	37	63	323	60	134	236	160	94	90	126
Sep	-	66	100	-	146	104	139		92	111	144	163	102	176	83	50	118
Oct	45	-	64	-	68	59	100		94	64	131	181	225	164	102	52	124
Nov	79	-	111	-	159	116	122	89		108	106	131	250	244	58	108	135
Dec	64	-	71	-	94	76	259	220	243	130	120	174	234	222	134	83	182
No. of samples	12	35	47	-	33	32	50	53	51	54	52	52	51	29	53	50	50
Average by month	63	90	123	-	104	104	182	176	157	172	153	185	241	198	115	145	171
by sample	63	76	102		119	88	173	155	131	157	161	178	244	204	106	117	163
Average Mar-Nov	62	60	94	-	105	85	127	118	111	146	123	155	233	195	86	123	139
Dec-Feb	64	300	213	-	107	174	346	310	282	249	242	266	263	222	202	213	262

Table S5. Summary of ambient monitoring data from the Ghanaian Environmental Protection Agency for 2017. Concentrations in µg/m^3. Source: Ghana EPA (see below).

	Permanent - PM10						Roadside PM10										
Month or Period	East Legon	Dansoman	North Industrial Area	South Industrial Area	Odorkor	5-site Average	First light	Shangri La	Achimota	La palm	Mallam market	Graphic	Weija	Kasoa	Tantra Hill	Amasaman	11-site Average
Jan	105	-	143	-	149	132	131	206	206	158	132	226	317	236	167	134	191
Feb	100	-	111	-	112	108	292	254	229	222	139	324	69	278	315	181	230
Mar	88	-	112	-	84	95	194	148	205	196	241	306	472	278	28	219	229
Apr	-	-	78	-	-	78	139	157	236	132	97	257	306	139	97	111	167
May	-	69	36	-	-	53	106	125	131	83	93	130	199	106	94	79	115
Jun	-	46	47	-	-	47	56	93	66	111	97	128	245	160	76	74	111
Jul	22	62	55	-	-	46	162	171	148	188	176	171	171	208	148	153	170
Aug	94	29	51	-	-	58	132	118	132	139	160	233	208	35	63	142	136
Sep	131	42	43	-	-	72	158	150	97	142	150	181	148	133	108	117	138
Oct	63	58	175	111	-	102	172	228	156	164	156	189	476	122	142	208	201
Nov	60	110	187	108	-	116	118	144	167	174	139	97	431	-	148	125	171
Dec	-	46	-	-	-	46	218	146	153	285	324	-	449	162	181	273	243
No. of samples	22	21	40	6	9	20	39	41	46	35	39	37	35	32	43	38	39
Average by month	83	58	94	110	115	85	157	162	161	166	159	204	291	169	131	151	175
by sample	83	58	122	76	114	80	157	162	161	166	159	187	291	155	131	163	173
Average Mar-Nov	76	59	87	110	84	78	137	148	149	148	145	188	295	148	100	136	160
Dec-Feb	103	46	127	-	131	109	214	202	196	222	198	275	278	225	221	196	221

In a visit to the EPA, we obtained 2017 data and some details on environmental monitoring, courtesy of Mr. Emmanuel Apoh (Head of Environmental Monitoring). The 2017 data is summarized in Table S3.

The EPA samplers were placed in cages at a height of ~5 m above ground level and at least 3.5 m from buildings, trees, and other obstacles, generally within an arc of 270-360 degrees to permit free air flow. 24-hr PM sampling took place every 6th day using the integrated samples collected on 47 mm diameter pore quartz filters at 5 L/min. The sampler type and other sampling and analytical details are unknown.

The 2017 PM_{10} data for the non-Harmattan season at the EPA sites in Accra and in this study are plotted in Figure S12. In this period, levels at the downwind site (190 µg/m^3) are nearly identical to the nearby Graphic Road site (188 µg/m^3); agreement diminished during the Harmattan period (186 and 275 µg/m^3, respectively), however, we had very few measurements at this site during this period..

In 2017, $PM_{2.5}$ sampling was limited, and only data for the Tantra Hill roadside site was made available (not shown). At this site, 41 samples were collected with an average concentration of 90 µg/m^3; the non-Harmattan and Harmattan periods averaged 82 and 113 µg/m^3, respectively, equivalent to 82 and 51% of PM_{10} measured at the same site. This ratio is similar to those in 2015 seen at the other roadside sites, noted previously.
